# NRIP1 disrupts ERα signal in Sjögren’s disease via AQP5 suppression and MYC-driven salivary dysfunction

**DOI:** 10.1038/s12276-026-01671-w

**Published:** 2026-03-13

**Authors:** Bo Chen, Janak L. Pathak, Xiuni Qin, Xueyang Li, Tianjiao Mao, Xi Chen, Wei Wei, Nobumoto Watanabe, Lijing Wang, Kevin H. Mayo, Jun Di, Yongliang Huo, Xiaomeng Li, Jiang Li

**Affiliations:** 1 School and Hospital of Stomatology, Guangdong Engineering Research Center of Oral Restoration and Reconstruction, Guangzhou Key Laboratory of Basic and Applied Research of Oral Regenerative Medicine, Guangzhou Medical University, Guangzhou, China; 2 Guangzhou Concord Cancer Center, Guangzhou, China; 3 Department of Dentistry and Endodontics, Affiliated Stomatology Hospital of Guangzhou Medical University, Guangzhou, China; 4 Hospital of Stomatology, Jilin University, Changchun, China; 5 Chemical Resource Development Research Unit, RIKEN Center for Sustainable Resource Science, Saitama, Japan; 6 Department of Biochemistry, Molecular Biology and Biophysics, University of Minnesota Health Sciences Center, Minneapolis, MN USA; 7 Beijing University of Chinese Medicine Hospital in Shenzhen, Shenzhen, China; 8 Guangzhou Municipal and Guangdong Provincial Key Laboratory of Protein Modification and Degradation, School of Basic Medical Sciences, Guangzhou Medical University, Guangzhou, China; 9 KingMed School of Laboratory Medicine, Guangzhou Medical University, Guangzhou, China

**Keywords:** Molecular biology, Cell biology

## Abstract

Sjögren’s disease (SjD) is marked by dysfunction of the salivary gland (SG) caused by epithelial cell death. However, the mechanism remains unclear. Here we discovered that NRIP1 was abnormally upregulated in SjD and formed a protein complex with estrogen receptor α (ERα) to inhibit saliva secretion and lead to epithelial cell death. NRIP1 interacted with ERα and altered the estradiol (E2)–ERα downstream signal in the SG epithelium. In the context of SjD, NRIP1–ERα suppressed aquaporin-5 (AQP5) expression and promoted MYC expression. The NRIP1–ERα complex bound to the estrogen response elements of the *AQP5* promoter, leading to the downregulation of AQP5 expression and reduced SG secretion. Conversely, the NRIP1–ERα complex bound to the estrogen response elements of the *MYC* promoter, resulting in the upregulation of MYC expression. Furthermore, we demonstrated that elevated MYC levels promoted apoptosis and altered immune regulation and cell metabolism in SjD. *Nrip1*-knockout/ovariectomized mice did not develop the SjD phenotypes, confirming the role of NRIP1 in the pathophysiology of SjD. Molecular dynamic simulations revealed that NRIP1 competitively bound to ERα and masked the E2 binding site, providing structural insights into the disruption of hormonal signal. This study implicates NRIP1 as a potent diagnosis parameter and provides a putative target for SjD management.

## NRIP1 complex impairs saliva secretion in Sjögren’s disease

This study explores Sjögren’s syndrome (SjD), a condition causing dry mouth and eyes, often affecting postmenopausal women. Researchers aimed to understand why salivary gland cells malfunction in SjD. The team found that a protein called NRIP1 is overactive in these cells during SjD. They used mouse models and cell experiments to study this. NRIP1 interacts with another protein, ERα, affecting two key genes: AQP5, which helps with saliva production, and MYC, which influences cell survival and immune response. In SjD, NRIP1 reduces AQP5, leading to less saliva, and increases MYC, causing cell damage. The researchers also discovered that removing NRIP1 in mice prevented SjD symptoms. This suggests that targeting NRIP1 could be a new way to treat SjD. Future research may focus on developing therapies that adjust NRIP1 activity to improve symptoms in patients with SjD.

This summary was initially drafted using artificial intelligence, then revised and fact-checked by the author.

## Graphical abstract

**Figure:**
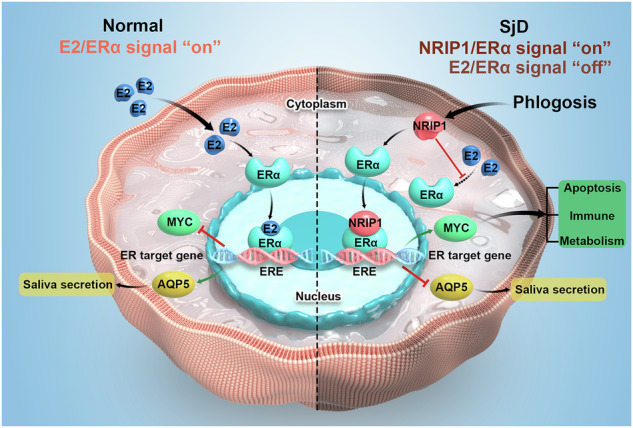
SjD-related phlogosis not only switches off the E2–ERα signal but also activates the NRIP1–ERα signal that downregulates AQP5 expression and upregulates MYC expression to aggravate SjD.

## Introduction

Sjögren’s disease (SjD) is an autoimmune inflammatory disease characterized by dryness, including the mouth and eyes, and is the most common in postmenopausal and older women^1,2^. The death and dysfunction of salivary gland (SG) epithelial cells (SGECs) in SjD lead to a decrease in saliva secretion^3^. The death of SGECs and degeneration of SGs release antigens Ro/SSA and La/SSB that initiate autoimmunity and systemic inflammation^4,5^. Classical estradiol (E2)–estrogen receptor α (ERα) signal has a substantial regulatory effect on SG function. As E2 maintains SGEC survival and saliva secretion, the reduced level of E2 in postmenopausal women is considered a contributing factor to SjD^6,7^. However, the mechanism of SGEC death and dysfunction in SjD remains unclear.

Aquaporin-5 (AQP5) is the key protein that plays a role in saliva secretion and is highly expressed in SGECs^8,9^. AQP5 is also involved in salivary development and function^10^. Studies have shown reduced expression and altered distribution of AQP5 in the SG of patients with SjD^11,12^. Lai et al. reported that AQP5 gene therapy restores AQP5 expression and saliva secretion in a mouse model of SjD^13^. Even though these reports indicate that SjD is characterized by an imbalance of AQP5, the mechanism of its downregulation is unclear. Furthermore, the proto-oncogene MYC regulates various cellular events related to immune dysfunction, cancer and neoplastic transformation^13–15^. Robust MYC expression has been observed in SGECs of patients with SjD^16,17^. Nevertheless, the regulation of MYC expression in SGECs and its role in SjD progression also remains unclear.

Both ERα and ERβ are highly expressed in SGECs and ductal epithelial cells, with AQP5 being a downstream target of E2–ERα signal^18,19^. The *AQP5* promoter has multiple high-scoring estrogen response elements (EREs) that respond to the ERα signal^20,21^. Both ovariectomized (OVX) and nonobese diabetic (NOD) mice exhibit the SjD phenotype and xerostomia^22,23^. Our previous work demonstrated that E2 or phytoestrogen apigenin restores SjD-downregulated AQP5 in SGECs and SG function in OVX mice^24,25^. These results indicate that the ERα signal is an upstream regulator of AQP5 expression in SGECs. Moreover, another phytoestrogen, quercetin, has been reported to promote epidermal stem cell proliferation via ER-mediated upregulation of MYC^26^. However, how ERα regulates AQP5 and MYC expression in SGECs when E2 levels are reduced in SjD is unclear.

Nuclear receptor-interacting protein 1 (NRIP1), also known as receptor-interacting protein 140 (RIP140), is an essential transcriptional coregulator^27^. NRIP1 plays a vital role in female reproduction, inhibition of mammary ductal differentiation, biorhythm, fat metabolism and tumor regulation^28–31^. On the basis of submandibular gland (SMG) transcriptomic analysis in SjD animal models, we found that NRIP1 is the sole regulatory transcription factor within the ERα signal, showing high expression levels in both patients with SjD and models, with diagnostic value, suggesting its potential involvement in the pathogenesis. Peters et al. reported that NRIP1 acts as a transcriptional corepressor of the classical E2–ERα signal^32^, while recent studies reveal it may also function as a co-activator of ERα signal^27,33^. In addition, NRIP1 is critical for the regulation of female reproduction and mammary gland architecture—estrogen signal is a central pathway governing ovulation and mammary gland development, and NRIP1 modulates gonadal development by inhibiting the classical ER signal^34,35^. Collectively, these studies indicate that NRIP1 plays a pivotal role in determining glandular developmental fate. However, the expression pattern of NRIP1 in SjD and its specific regulatory role in the ERα signal remain to be fully elucidated.

Here, we investigate a regulatory mechanism involving the NRIP1–ERα axis in the dysfunction of SGECs during SjD. We found that NRIP1 is upregulated in SGECs during SjD and interacts with ERα in these cells owing to a deficiency of E2. The NRIP1–ERα complex binds to ERE sites on the *AQP5* promoter, inhibiting AQP5 expression and saliva secretion. Furthermore, this complex also binds to ERE sites on the *MYC* promoter, leading to the upregulation of MYC expression. Elevated MYC levels promote apoptosis in SGECs, thereby affecting immune regulation and cellular metabolism. Notably, *Nrip1*-knockout (KO)/OVX mice resulted in the absence of SjD phenotype development. In addition, computational simulations suggest that NRIP1 may exert a competitive inhibition effect against E2–ERα signal. Overall, these findings underscore a specific regulatory relationship among NRIP1, ERα, AQP5 and MYC, indicating that targeting the NRIP1–ERα signal axis may represent a promising therapeutic strategy for SjD.

## Materials and methods

### Chemicals and reagents

The following reagents used in this study were purchased from the following companies: E2 (Baoji Herbest Bio-Tech); IFN-γ (Guangzhou Ruigan Bio-Tech); fulvestrant (MCE); fetal bovine serum (FBS; Angke Information Technology); 1640 medium (Onco Information Technology); total RNA extraction kit (Yeasen Biotechnology); real-time quantitative PCR (RT–qPCR) kit (Accurate Biotechnology); RT–qPCR primers (Sangon Biotech); mouse anti-AQP5 (mouse monoclonal, Santa Cruz Biotechnology); mouse anti-ERα (mouse monoclonal, Santa Cruz Biotechnology); mouse anti-NRIP1 (mouse monoclonal, Santa Cruz Biotechnology); rabbit anti-MYC (rabbit polyclonal, ABclonal); mouse anti-BAX (rabbit polyclonal, Bioss); mouse anti-LTF (rabbit polyclonal, Bioss); rabbit anti-CASP7 (rabbit polyclonal, Bioss); rabbit anti-IKBKG (rabbit polyclonal, Bioss); rabbit anti-PLA2G7 (rabbit polyclonal, Bioss); rabbit anti-RCN1 (rabbit polyclonal, Bioss); rabbit anti-DAB2IP (rabbit polyclonal, Bioss); rabbit anti-CFLAR (rabbit polyclonal, Bioss); rabbit anti-HMG20A (rabbit polyclonal, Bioss); rabbit anti-S100A1 (rabbit polyclonal, Bioss); rabbit anti-GLANT4 (rabbit polyclonal, Bioss); rabbit anti-STX4 (rabbit polyclonal, Solarbio); rabbit anti-GAPDH (mouse monoclonal, Proteintech); horseradish peroxidase (HRP)–coupled secondary antibody, including anti-rabbit IgG and anti-mouse IgG (Bioss); luciferase reporter detection reagents (Promega); co-immunoprecipitation (Co-IP) Assay Kit (Beyotime); and Chromatin Immunoprecipitation (ChIP) Assay Kit (ABclonal).

### Animal studies

This study used female Institute of Cancer Research (ICR) mice (29 ± 4 g) and NOD mice (22 ± 3 g) aged 6–8 weeks, sourced from Hua Fukang Biotechnology, which served as the SjD model and control groups, respectively^24,36^. Similarly, clear protocols for sham and OVX mice were also provided in our earlier studies^24^. *Nrip1*-KO mice were purchased from Cyaye Biotechnology. *Nrip1* is located on chromosome 16 of mice. Using CRISPR–Cas technology, single guide RNA is designed to target *Nrip1*, leading to the generation of *Nrip1* gene KO mice through high throughput electrofertilization of zygotes. Sperm samples were collected and cryopreserved after sexual maturity. After the observation period, mice were anesthetized and SMG tissue was extracted for RT–qPCR, Co-IP, immunofluorescence and immunohistochemistry analyses. RNA sequencing (RNA-seq) and ChIP sequencing (ChIP-seq) were then conducted on the extracted SMG tissue.

### RNA-seq, scRNA-seq and microarray data

SMG tissue was extracted from mice, and total RNA was isolated using Trizol reagent. The RNA samples underwent thorough quality control before messenger RNA enrichment via a polyA tail using Oligo (dT) magnetic beads. The mRNA was then randomly fragmented using divalent cations in NEB Fragmentation Buffer. Library construction was performed using either the NEB ordinary or strand-specific methods. Initial quantification was conducted with a Qubit 2.0 fluorometer, while the insert size was determined using an Agilent 2100 bioanalyzer, and the effective concentration was accurately quantified through RT–qPCR. Sequencing of all transcribed mRNAs from the tissue was executed on the HiSeq platform, following the Illumina TruSeq RNA sample preparation kit protocol. In addition, single-cell RNA sequencing (scRNA-seq) data (accession no. HRA003613) were obtained from the Genome Sequence Archive of the National Genomics Data Center. This dataset includes samples from 11 patients with SjD and 5 normal control samples. The Seurat R software package was used to identify and interpret the heterogeneous sources of scRNA-seq data, as well as to integrate various types of single-cell data. Microarray data for SjD were downloaded from the GEO database (http://www.ncbi.nlm.nih.gov/geo/). These three datasets are derived from different General Public License-covered platforms, which enhances the reliability of the study and mitigates the limitations associated with a single dataset or platform. For the analysis of target molecules, we used the linear model from the LIMMA package to examine differences in expression values among samples. Information regarding the microarray datasets is presented in Supplementary Table 1.

### ChIP-seq

SMG tissues from ICR and NOD mice were used for the ChIP assay, using a rabbit polyclonal anti-ERα antibody obtained from Abcam. The ChIP samples were processed following the instructions provided by Illumina. After qualification testing of the library, DNA libraries were sequenced using the Illumina NovaSeq 6000 platform. The ChIP-seq process comprised raw read processing, filtering, delinking, decontamination, alignment to the reference genome and analysis of high-quality mapped reads (MAPQ ≥30).

### Bioinformatics analysis

To enhance the critical data obtained from RNA-seq, scRNA-seq and microarray datasets, we conducted a series of bioinformatics analyses. These analyses included gene set enrichment analysis (GSEA) using the MSigDB database through the JAVA program to investigate the biological processes influenced by gene expression within the dataset; univariate analysis executed with the linear model of the LIMMA package to identify statistically significant differentially expressed genes (DEGs) on the basis of expression value differences between samples; using the DAVID (http://david.abcc.ncifcrf.gov/) for gene analysis and the assessment of gene biological functions; evaluating the diagnostic validity of receiver operating characteristic (ROC) analysis indicators using the pROC package; utilizing STRING (https://string-db.org) to retrieve interactions among genes and proteins; and analyzing scRNA-seq data with R and the Seurat package.

### Patients with SjD and healthy individuals

Minor SG biopsies were collected from patients referred to the Affiliated Stomatology Hospital of Guangzhou Medical University for suspected SjD. SjD is defined according to the 2016 criteria established by the American College of Rheumatology and the European League Against Rheumatism, as presented in Supplementary Table 2. Individuals with a score of >4 are classified as having SjD^37,38^. The minor SG biopsies were used for immunohistochemical and immunofluorescence analyses of the NRIP1, ERα, AQP5 and MYC proteins. The detailed clinical data of the five patients with SjD included in this study are presented in Supplementary Table 3.

### Cell culture and transfection

For this study, we used the A253 epithelial cell line^39–41^ that was obtained from Zhejiang Meisen Cell Biotechnology. Cells were cultured in Roswell Park Memorial Institute (RPMI) 1640 medium supplemented with 10% FBS, 2 mM/l glutamine, 100 U/ml penicillin and 100 μg/ml streptomycin. The cells were incubated at 37 °C, 5% CO_2_ and saturated humidity. Passage was usually performed every 2–3 days, and cells in the logarithmic growth phase were used for experiments.

Transfection of small interfering (si)RNA or plasmids at working concentrations using lipofectamine 2000 reagent (Invitrogen) was performed according to the manufacturer’s protocol. sh-MYC plasmid was purchased from GenePharma, and siNRIP1 and NRIP1 overexpression plasmids were designed and synthesized by Tsingke Biological Technology. The target sequences of plasmids are presented in Supplementary Table 4.

### Immunohistochemical assay

SMG tissue was prepared in 5-μm sections that were dewaxed in xylene and hydrated in an ethanol gradient. After rinsing with distilled water, endogenous peroxidase activity was blocked by using a hydrogen oxide solution. Tissue sections were combined with primary antibodies (ERα (1:100), NRIP1 (1:200), AQP5 (1:100), MYC (1:200), BAX (1:100), CASP7 (1:100), IKBKG (1:100), STX4 (1:200), PLA2G7 (1:100), RCN1 (1:100), DAB2IP (1:100), CFLAR (1:100), HMG20A (1:100), S100A1 (1:100), LTF (1:100) and GLANT4 (1:100)) overnight at 4 °C. The tissue sections were then incubated with goat anti-rabbit or goat anti-mouse IgG antibody (1:500) for 1 h at room temperature. The sections were dehydrated in a fractionated ethanol series and sealed with neutral resin. Finally, the positively stained cells were observed under a light microscope. We analyzed the immunohistochemistry image data using ImageJ, obtaining the cumulative optical density by summing the optical density values of each brown dot within the image. This cumulative optical density was divided by the area of the effective target distribution to calculate the average optical density, which was subsequently compared between groups.

### Co-IP assay

Prepared SMG tissue and cell samples were lysed with ice-cold lysis buffer (Beyotime Biotechnology). The target protein-specific antibody with protein G-agarose beads (Beyotime Biotechnology) was incubated for 4 h at 4 °C and then the lysate was added. The mixture was incubated overnight at 4 °C. After three washes with cold PBS, the Co-IP products were collected, and the proteins were analyzed using western blotting.

### Immunofluorescence staining

Treated A253 epithelial cells were fixed with 4% paraformaldehyde for 15 min, followed by permeabilization with 0.1% Triton X-100 for 10 min. They were then blocked using 5% bovine serum albumin for 30 min at room temperature. Primary antibodies (ERα (1:100), NRIP1(1:200), AQP5 (1:100) and MYC (1:200)) were applied overnight at 4 °C. Donkey anti-rabbit Alexa Fluor 594–conjugated secondary antibody (1:500, ThermoFisher Scientific) and donkey anti-mouse Alexa Fluor 488–conjugated secondary antibody (1:500, ThermoFisher Scientific) were used. In addition, DAPI (1:1000, ThermoFisher Scientific) was used. Quantification on representative images at 60× magnification was performed by a blinded, independent observer.

### Fluorescence staining for YFP-H148Q-V163S

pcDNA3.1-YFP-H148Q-V163S eukaryotic expression vector was constructed using the point mutation kit, and lipofectamine 2000 reagent-mediated YFP-H148Q-V163S transfection of A253 epithelial cells treated with corresponding conditions to detect the water permeability (representing water secretion at the cellular level), which provides evidence of the functional activity of AQP5.

### RT–qPCR

The Trizol kit extracts total RNA from SMG tissue or cells. RNA was reverse transcribed to complementary DNA using the PrimeScriptTM RT kit (Takara Clontech). RT–qPCR was performed on the CFX Connect real-time system (BioRad) using the SuperReal PreMix Plus (SYBR Green) kit (Takara Clontech). The thermal cycling conditions for PCR amplification were 9 °C for 5 min, 95 °C for 10 s, 60 °C for 30 s, 40 cycles and then 40 °C for 20 min. mRNA expression was normalized to β-actin mRNA levels. Three separate experiments were conducted. Primers for RT–qPCR are presented in Supplementary Table 5.

### Western blotting

Total protein was extracted with RIPA lysis buffer (Beyotime); thereafter, the lysate was collected and centrifuged at 4 °C (12,000 rpm, 20 min). Proteins were separated and transferred to polyvinylidene difluoride membranes on 10% or 12% SDS–polyacrylamide gel electrophoresis gel (Beyotime), blocked in 5% skim milk for 1 h and incubated overnight with primary antibodies at 4 °C. After three washes with TBST buffer, we incubated the membrane-coupled secondary antibody with HRP for 1 h at room temperature. The bound antibodies were then detected using the Odyssey infrared imaging system (Li Biosciences).

### Dual-luciferase reporter assay

A253 epithelial cells were seeded in six-well plates with cells from each well transfected with 2 µg *AQP5*p-luc/*MYC*p-luc plasmid and 2 µg NRIP1 overexpression plasmid for 48 h. Cells were lysed in 500 µl lysis buffer with shaking on ice for 30 min. After adequate lysis, the lysate was transferred to a new tube, centrifuged at 15,000*g* for 5 min and the supernatant was used for the assay. For the firefly luciferase activity assay, 50 µl supernatant was used in a dual-luciferase reporter assay system. The ratio of firefly luciferase activity to sea cucumber luciferase activity was calculated as relative luciferase activity.

### ChIP assay

The chromatin solution was sonicated and incubated with anti-ERα and swirled overnight at 4 °C. DNA–protein crosslinks are reversed, and chromatin DNA is purified and subjected to PCR analysis. After amplification, PCR products are separated on a 2% agarose gel. The hTFtarget database predicted *AQP5* and *MYC* promoter regions containing ERα binding sites, respectively. The following specific primers were used for PCR: *AQP5* promoter (forward primer: 5’-TTGGGAGGTCAGTGGTGC-3’, reverse primer: 5’-TGGAAGGCTGGCGTTTT-3’) and *MYC* promoter (forward primer: 5’-TTCCTCCAGTAACTCCTCTT-3’, reverse primer: 5’-AAAGTAAGTGTGCCCTCTAC-3’).

### Molecular docking experiments

The Protein Data Bank (PDB) file of the target gene was obtained from the RCSB PDB online database (https://www.rcsb.org/). Water molecules and duplicate entries were removed using PyMOL software, followed by the addition of polar hydrogen atoms. Subsequently, the file was converted to PDBQT format using AutoDock and saved. The AutoGrid 4 parameter configuration file was initialized, and the file was imported. The grid spacing was set to the predetermined value, and the lattice center coefficient was adjusted to ensure complete coverage of the recipient protein. The AutoGrid 4 program was then executed. The Dock parameter file was set with default values for the remaining parameters, and the docking parameter file was exported. Finally, the AutoDock 4 program was executed once more. On the basis of the analysis provided by AutoDock 4, the binding energy between proteins was calculated to identify all amino acids involved in the protein–protein interactions. Visualization and further analysis were conducted using PyMOL software.

### Statistical analysis

All data are expressed as the mean ± standard deviation (s.d.). Data were analyzed statistically by using a two-tailed Student’s *t*-test or one-way analysis of variance by post hoc comparison. Graphs were generated using GraphPad 9. Each experiment was repeated at least three times. The difference was considered statistically significant at *P* < 0.05.

## Results

SjD exhibits two critical characteristics, that is, primary estrogen decline during aging and secondary inflammatory damage. We used two SjD mouse models. The first model was NOD mice. As a model of inflammation, NOD mice display significant inflammatory damage in the SMG, leading to dry mouth and systemic symptoms of dehydration^42^. The second model was OVX mice that mimic primary SjD during female menopause. The deprivation of estrogen in these mice leads to degenerative changes in SMG, causing dry mouth and systemic dehydration^23^. Our previous findings demonstrated a significant xerostomia phenotype in animals. In comparison to the ICR group, the NOD group exhibited reduced saliva secretion, increased water consumption, decreased acinar destruction, inflammatory infiltration and AQP5 expression in SMG^36^. Similarly, the OVX group showed a significant decrease in saliva secretion, increased water consumption and decreased AQP5 expression in the SMG compared with the control group^24^. These results strongly support the reliability of our experimental mouse models.

### NRIP1 expression is elevated in SjD SGs

**Fig. 1:**
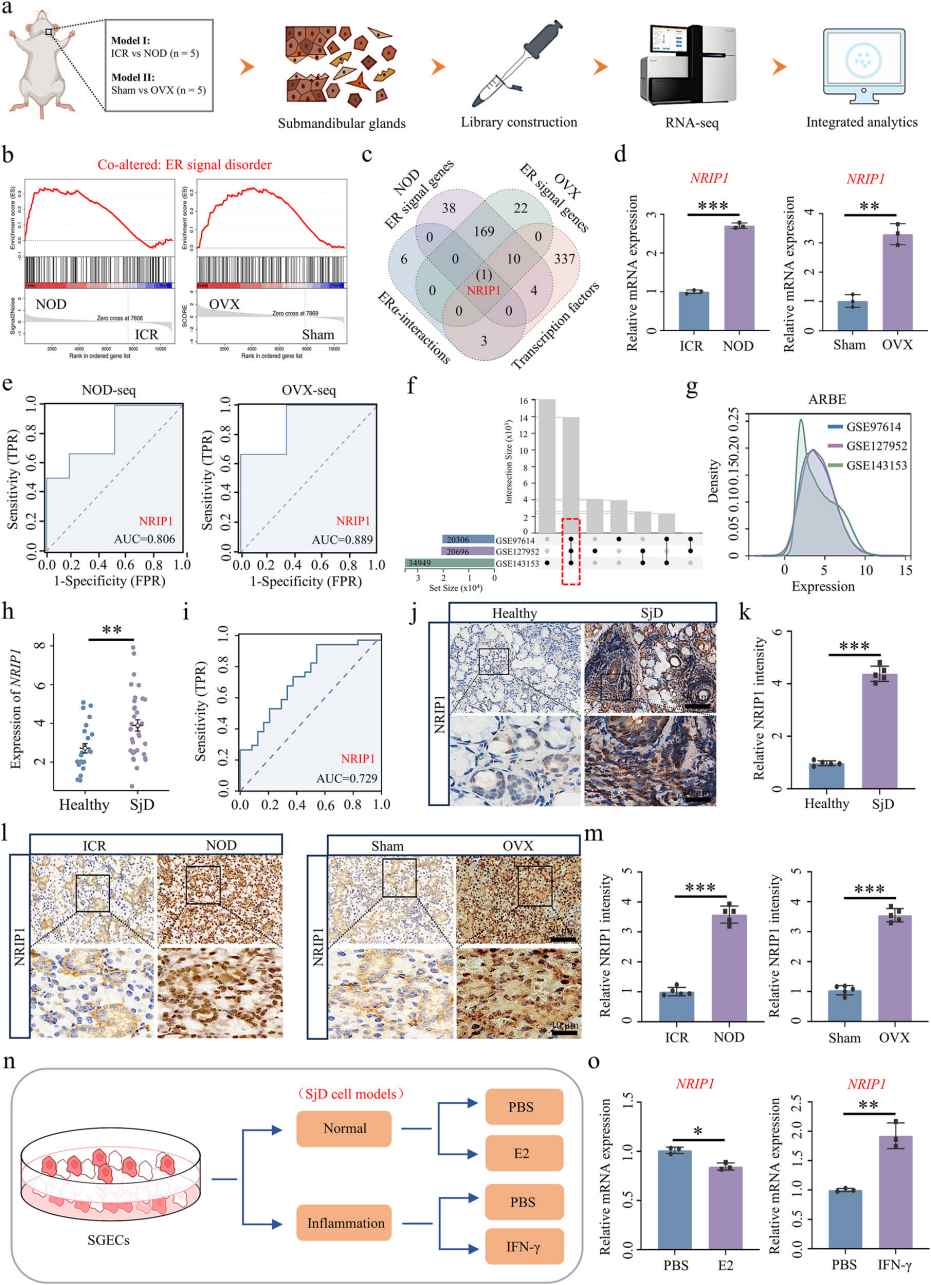
NRIP1, a core ERα signal regulatory gene, is upregulated in SjD pathogenesis. **a** An overview of the workflow used in RNA-seq experiments. **b** GSEA reveals alterations in ER signal within the SMG of both NOD and OVX mice. **c** The core gene, *NRIP1*, was identified through a systematic screening process. **d** The mRNA expression levels of *NRIP1* in the SMG of SjD mouse models were quantified using RT–qPCR (*n* = 3). **e** ROC analysis was conducted for diagnostic purposes. **f** The UpSet analysis was used to identify co-expressed genes across three distinct SjD datasets and to integrate the samples. **g** Density maps show the effects of combining multiple SjD SG transcriptome datasets following batch effect removal. **h** A scatter plot demonstrates a reduction in *NRIP1* expression in SjD. **i** Diagnostic analysis of NRIP1 in SjD was performed. **j**, **k** Immunohistochemical methods were used to detect NRIP1 in SjD SGs (*n* = 5). **l**, **m** Immunohistochemical detection of NRIP1 was also conducted in SMG tissue from the SjD mouse models (*n* = 5). **n** The construction scheme for the SjD cell models is depicted. **o** The mRNA expression of *NRIP1* in SjD cell models was assessed using RT–qPCR (*n* = 3). ARBE, after remove batch effect; Sham, sham surgery.

To further investigate the potential mechanisms underlying the pathogenesis of SjD, we conducted RNA-seq on SMG from two SjD mouse models, namely, the NOD and OVX models (Fig. 1a). Through GSEA, we identified the ER signal as the most critical disorder pathway in both models (Fig. 1b). This finding suggests a close association between ER signal disorders and the development of SjD. We screened the commonly annotated genes related to ER signal and retrieved ten highly interactive ERα-interacting genes from the STRING, alongside 355 transcription factors documented in the hTFtarget database. Notably, NRIP1 was the only gene overlapping across all four datasets (Fig. 1c). We performed RT–qPCR on the SMG tissues from these two SjD mouse models, and the results demonstrated that *NRIP1* was significantly upregulated in both the NOD and OVX models compared with their respective control groups (Fig. 1d). Furthermore, ROC analysis revealed that the area under the curve values for NRIP1 in the NOD and OVX models were 0.806 and 0.889, respectively (Fig. 1e), indicating strong diagnostic potential for NRIP1 in both SjD mouse models. On the basis of these findings, we identified NRIP1 as a core modulator of SjD.

On the basis of our findings, we explored three SjD datasets from the GEO database, which included SG tissues from both patients with SjD and healthy controls. Through Upset analysis, we identified co-expressed genes across the three datasets and subsequently integrated the samples (Fig. 1f). The ComBat function was then used to eliminate batch effects, facilitating a more robust comparative analysis. Density plots showed that the data distribution became consistent across datasets following batch effect correction (Fig. 1g and Supplementary Fig. 1). Our results indicated that *NRIP1* was significantly upregulated in the patients with SjD compared with healthy controls (Fig. 1h). ROC analysis further validated the diagnostic potential of NRIP1 expression levels for SjD, yielding an area under the curve of 0.729 (Fig. 1i). Immunohistochemical staining of clinical SjD samples demonstrated that NRIP1 was markedly overexpressed and translocated into the nucleus in the SGs of patients with SjD compared with healthy controls (Fig. 1j,k). These findings suggest that NRIP1 expression is significantly elevated during the progression of SjD.

To further validate the expression of NRIP1 in SjD, we conducted additional studies using the previously mentioned mouse models. Immunohistochemical analysis of SMG tissues from SjD mouse models revealed that NRIP1 was significantly overexpressed and localized to the nucleus in the model groups when compared with their respective control groups (Fig. 1l,m). Furthermore, we established two in vitro cell models: an E2-induced physiological model simulating normal estrogen status and an IFN-γ-induced inflammatory model simulating the inflammatory conditions characteristic of SjD (Fig. 1n). We examined NRIP1 expression in these models. The results from RT–qPCR indicated that *NRIP1* transcript levels were slightly reduced under E2-induced physiological conditions but significantly increased under IFN-γ-induced inflammatory conditions (Fig. 1o). This suggests that the upregulation of NRIP1 occurs in the pathological contexts of SjD. In summary, NRIP1 overexpression in SGs is a common feature observed in SjD models and its elevation represents a critical pathological hallmark of the disease.

### NRIP1 interacts and modulates ERα functions in SjD pathogenesis

**Fig. 2:**
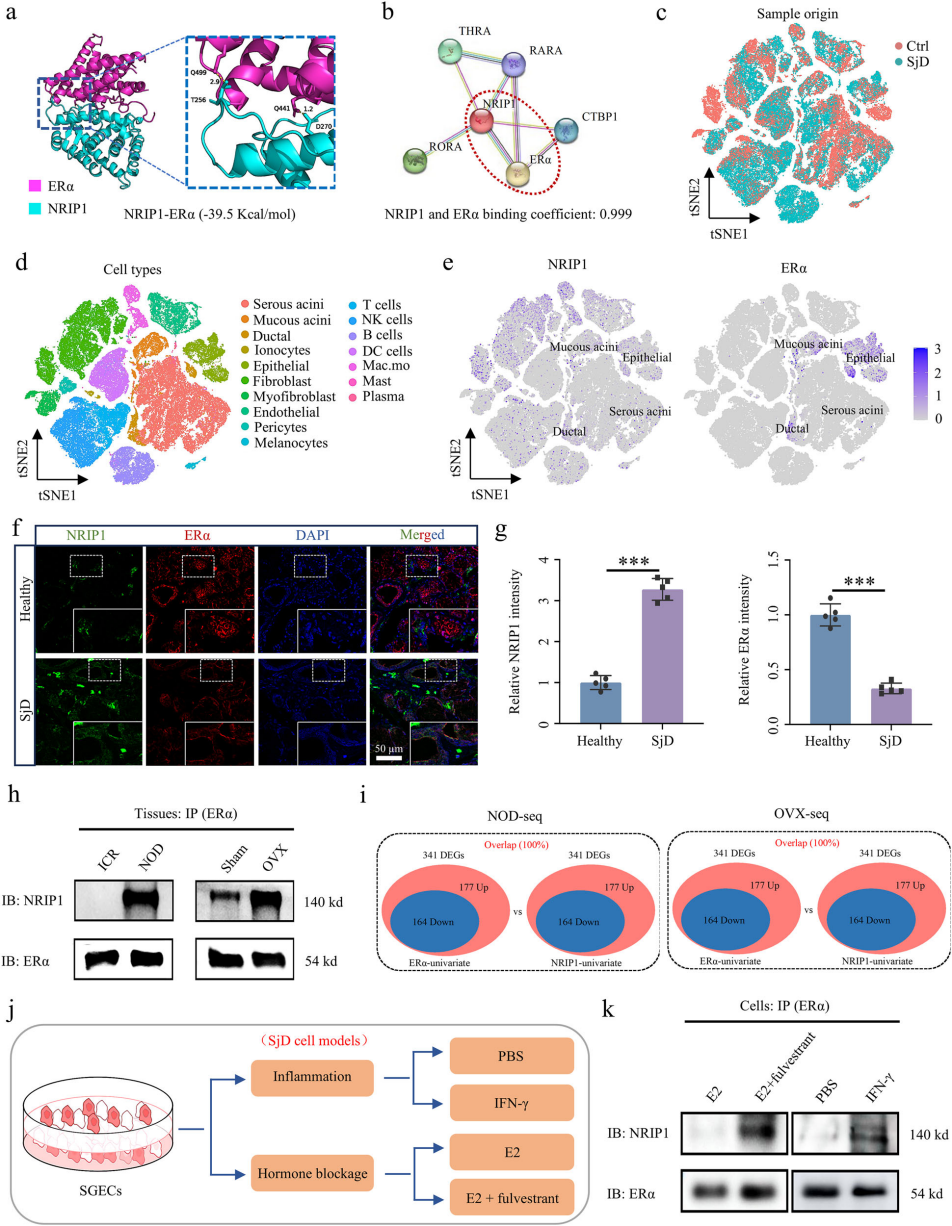
NRIP1 interacts with ERα and shares regulatory functions in SjD lesions. **a** The structural model of NRIP1–ERα. **b** According to the STRING database, NRIP1 exhibits the strongest interaction with ERα, characterized by a binding coefficient of 0.999. **c**, **d** The t-SNE plot (**c**) shows the distribution of cells in SG tissues, colored by sample origin, while **d** shows the cell subset annotation. **e** Gene expression levels of NRIP1 and ERα in specific cells are presented. **f**, **g** Immunofluorescence and quantitative analysis of clinical samples from SjD patients revealed co-localization of NRIP1 and ERα in SG tissues (*n* = 5). **h** The interaction between NRIP1 and ERα was confirmed using a Co-IP assay in the SMG tissue of an SjD mouse models. **i** On the basis of transcriptomic data from SjD mouse models, univariate analysis of NRIP1 and ERα was conducted, revealing that the downstream DEGs regulated by both NRIP1 and ERα were 100% coincident in SjD models. Nod-seq, NOD versus ICR; Ovx-seq, OVX versus sham. **j** The construction scheme of the SjD cell models is shown. **k** Using the SjD cell models, Co-IP assays demonstrated the interaction between NRIP1 and ERα. Significant difference, ^***^P < 0.001. Sham, sham surgery. Panel **a** created with AlphaFold.

NRIP1 functions as a transcriptional modulator of ERα signal. However, the association between NRIP1 and specific diseases remains inadequately explored, particularly with regard to SjD, which has not been previously reported. Molecular docking analysis using AlphaFold indicated a significant binding energy of −39.5 kcal/mol between ERα and NRIP1 (Fig. 2a). A search conducted in the STRING identified ERα as the most potent interactor with NRIP1, with a binding coefficient of 0.999 (Fig. 2b). This finding underscores a substantial interaction between NRIP1 and ERα. To evaluate gene expression at the cellular level, we retrieved scRNA-seq data from patients with SjD for analysis. Initially, cells were sorted according to disease states to investigate the distribution of cell types across different conditions (Fig. 2c). Cluster analysis using t-distributed stochastic neighbor embedding (t-SNE) revealed 17 distinct cell types, including serous acini, mucous acini, monocytes, macrophages, fibroblasts, myofibroblasts, pericytes, melanocytes, ductal cells, epithelial cells, endothelial cells, mast cells, plasma cells, T cells, B cells, natural killer cells and dendritic cells (Fig. 2d). The relative proportions of these cells across various disease states illustrated their distribution (Supplementary Fig. 2a). Gene expression patterns were visualized within the cell atlas, revealing concentrated expression of ERα genes predominantly in mucous acinar cells, epithelial cells and ductal cells. By contrast, NRIP1 exhibited a dispersed expression pattern across mucous acinar cells, epithelial cells and ductal cells (Fig. 2e and Supplementary Fig. 2b). These findings suggest that NRIP1 and ERα coexist within SGECs.

To confirm the interaction between NRIP1 and ERα, we conducted immunofluorescence staining on clinical samples from patients with SjD. The results indicated that, compared with the healthy group, the expression of NRIP1 in the SGs of patients with SjD was significantly enhanced and it colocalized with ERα in the SG tissues (Fig. 2f, g). Furthermore, we selected the SMG tissue from the aforementioned SjD mouse models and used an ERα antibody to immunoprecipitate the cosedimented proteins, followed by western blot analysis. The results demonstrated that NRIP1 protein was detectable in the ERα complex isolated from both NOD and OVX models, while the signal for NRIP1 was weak in the ICR and the sham groups, suggesting the presence of the NRIP1–ERα complex in the SjD mouse models (Fig. 2h). In addition, on the basis of the RNA-seq data from the aforementioned SjD mouse models, we performed a univariate analysis of NRIP1 and ERα, revealing that the downstream target genes regulated by both completely overlapped in the NOD model. Interestingly, the downstream target genes regulated by ERα and NRIP1 in the OVX model also exhibited a high degree of uniformity (Fig. 2i), indicating a regulatory function between NRIP1 and ERα.

In addition, we used SGECs to establish two in vitro models of SjD SG injury. The first model simulates an inflammatory response, induced by the addition of IFN-γ at a concentration of 100 ng/ml for 48 h^36^, which triggers SjD-related inflammation. The second model mimics the pathological state of SjD characterized by decreased estrogen levels, achieved through cotreatment with E2 at 10 nM and the ER antagonist fulvestrant at 1 μM (Fig. 2j). These models were used to investigate the interaction between NRIP1 and ERα under conditions analogous to SjD SG injury. Co-IP assays were conducted in the SjD cell models, revealing that NRIP1 was present in the ERα complex under conditions that mimic SjD damage, whereas only weak signals of NRIP1 were observed in the E2 and PBS groups. This finding suggests that the NRIP1–ERα complex emerges in states of inflammation or estrogen deficiency (Fig. 2k), consistent with tissue-level observations (Fig. 2h). Collectively, these results substantiate the colocalization of NRIP1 and ERα in patients with SjD, animal models and cell models, as well as their interaction and regulatory effects.

### *Nrip1*-KO/OVX mice fail to develop SjD phenotype

**Fig. 3:**
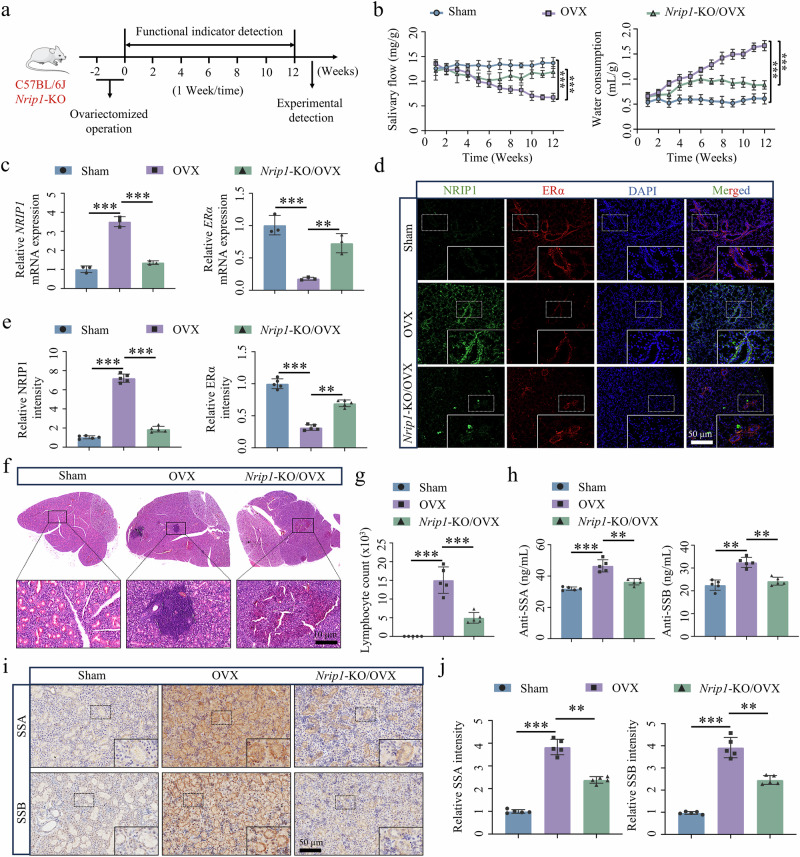
Knockdown of NRIP1 effectively corrected the SjD phenotype of OVX mice. **a** Scheme of animal experiment. **b** Saliva secretion and water consumption were measured in mice (*n* = 5). **c** *NRIP1* and *ERα* mRNA expression pattern in SMG tissue of mice (*n* = 3). **d**, **e** NRIP1 and ERα immunofluorescence and quantification of mouse SMG (*n* = 5). **f**, **g** H&E staining and quantification of mouse SMG (*n* = 5). **h** Detection of anti-SSA and anti-SSB antibody levels in the serum of mice (*n* = 5). **i**, **j** SSA and SSB immunohistochemistry and quantification of mouse SMG (*n* = 5). Significant difference, ^**^P < 0.01 and ^***^P < 0.001. Sham, sham surgery.

*Nrip1*-KO/OVX mice were generated to investigate the impact of *Nrip1* KO on SjD development (Fig. 3a). This study included three groups: sham, OVX and *Nrip1*-KO/OVX. After 12 weeks, we observed that saliva secretion in the OVX group decreased to 49.1% and water consumption increased to 273.8% of those in the sham mice. By contrast, mice in the *Nrip1*-KO/OVX group showed similar levels of saliva secretion (86.7% of sham group) and water consumption (145.1% of sham group) to the sham control group (Fig. 3b). We assessed the expression levels of NRIP1 and ERα in SMG tissue. Our RT–qPCR results indicated that *NRIP1* levels were significantly elevated in the OVX group, whereas *ERα* levels were reduced compared with the sham group. In the *Nrip1*-KO/OVX group, *ERα* expression returned to a level comparable to that of the sham group when *NRIP1* is absent (Fig. 3c). These findings corroborated the results obtained from immunofluorescence staining (Fig. 3d, e). Further, we observed significant alterations in the SMG and spleen indices (Supplementary Fig. 3a). Hematoxylin and eosin (H&E) staining of mouse SMG tissue revealed lymphatic infiltration foci in the OVX group compared with the sham group. Notably, the *Nrip1*-KO/OVX group exhibited a marked improvement in lymphatic infiltration within the SMG tissue (Fig. 3f,g). The presence of anti-SSA/SSB antibodies is a crucial clinical marker for SjD diagnosis. Evaluation of the therapeutic impact of *Nrip1* KO involved eye blood respiration and enzyme-linked immunosorbent assay analysis using isolated serum. Results indicated a significant increase in serum anti-SSA and anti-SSB antibody levels in the OVX group. Conversely, serum levels of anti-SSA and anti-SSB antibodies in the *Nrip1*-KO/OVX group returned to normal levels (Fig. 3h). Consistent outcomes were also observed in the analysis of SSA and SSB antigens in mouse SMG tissue (Fig. 3i, j and Supplementary Fig. 3b). These findings suggest that downregulation of NRIP1 effectively reduces anti-SSA and anti-SSB antibodies in the serum of OVX mice. In summary, these findings demonstrate that OVX mice develop SjD phenotype but *Nrip1*-KO reversed these phenotyps in OVX mice, suggesting a critical role of NRIP1 in SjD development and progression.

### The NRIP1–ERα complex correlates negatively with AQP5 and positively with MYC

**Fig. 4:**
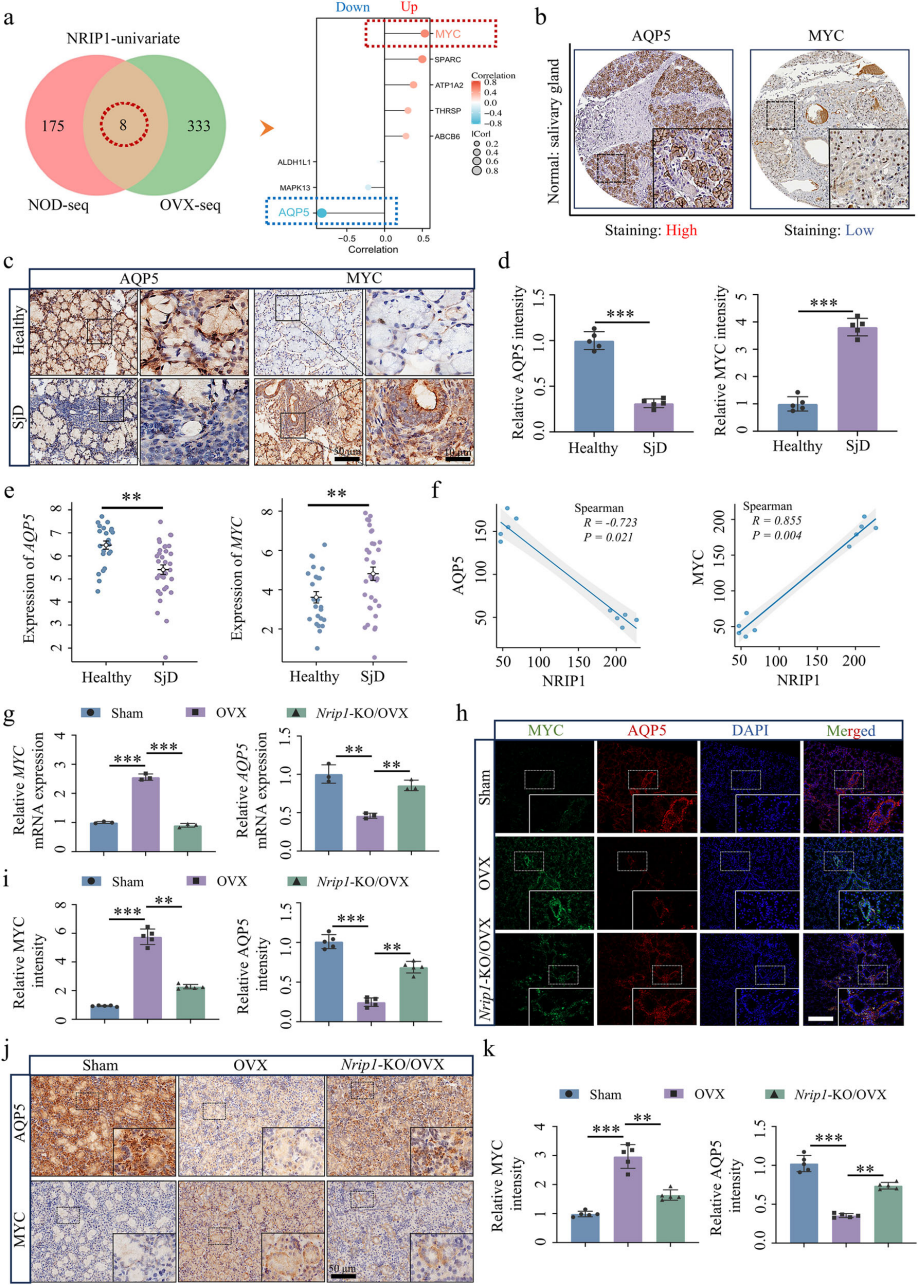
The NRIP1–ERα complex was negatively correlated with the AQP5 and positively associated with MYC. **a** RNA-seq data-based NRIP1 univariate analysis of overlapping DEGs in NOD and OVX mice SMGs and correlation analysis of NRIP1 and overlapping genes. **b** Images of healthy human SG obtained from the HPA database demonstrate a higher expression of AQP5 and a lower expression of MYC. **c**, **d** Immunohistochemical staining and quantification of SjD clinical samples (*n* = 5). **e** Scatter plot showing that immunohistochemical staining of SjD clinical samples decreased *AQP5* expression while *MYC* expression increased in SjD. **f** On the basis of quantitative immunohistochemical analysis of NRIP1, AQP5 and MYC in SjD SGs, Spearman analysis was used to assess the correlation between NRIP1 and AQP5 or MYC (*n* = 5). **g** *MYC* and *AQP5* mRNA expression pattern in SMG tissue of mice (*n* = 3). **h**–**k** MYC and AQP5 immunofluorescence (**h** and **i**) and immunohistochemistry (**j** and **k**) in the SMG of mice (*n* = 5). Significant difference, ^**^P < 0.01 and ^***^P < 0.001. Sham, sham surgery.

To investigate the regulatory mechanism of the NRIP1–ERα complex in SG function, we used univariate analysis on NRIP1 with RNA-seq data from our two mouse models of SjD. This analysis identified eight DEGs that were common in both models. Among these, AQP5, MAPK13 and ALDH1L1 negatively correlated, whereas MYC, SPARC, ATP1A2, THRSP and ABCB6 positively correlated with NRIP1–ERα (Fig. 4a). Among these eight genes, NRIP1 showed the strongest negative correlation to AQP5 and MYC showed the strongest positive correlation (Fig. 4a). The Human Protein Atlas (HPA) database was used to examine the expression of AQP5 and MYC in normal human SG tissue, revealing high AQP5 expression and low MYC expression (Fig. 4b). Compared with samples from healthy controls, immunohistochemical staining of SjD clinical samples further validated these findings by showing elevated nuclear MYC expression with significantly reduced AQP5 expression (Fig. 4c, d). The analysis of the human RNA-seq data described above showed that the expression level of *AQP5* was significantly reduced in the SjD group and *MYC* was significantly increased compared with the healthy control group (Fig. 4e). Spearman correlation analysis showed a significant positive correlation between NRIP1 and MYC and a significant negative correlation between NRIP1 and AQP5 (Fig. 4f). Furthermore, *Nrip1*-KO mice were used to evaluate the changes in the expression levels of MYC and AQP5 in SMG tissues. Our RT–qPCR results demonstrated that the OVX group exhibited significantly increased levels of *MYC*, whereas it demonstrated decreased levels of *AQP5*, compared with the sham group. In the *Nrip1*-KO/OVX group, expression of *MYC* was decreased and approached levels similar to the sham group, whereas *AQP5* expression returned to levels comparable to the sham group (Fig. 4g). These findings support those data from immunofluorescence and immunohistochemical assays (Fig. 4h–k). These findings suggest a role of the NRIP1–ERα complex in downregulating AQP5 and upregulating MYC in SjD.

### The NRIP1–ERα complex downregulates the AQP5 gene that is essential for saliva secretion

**Fig. 5:**
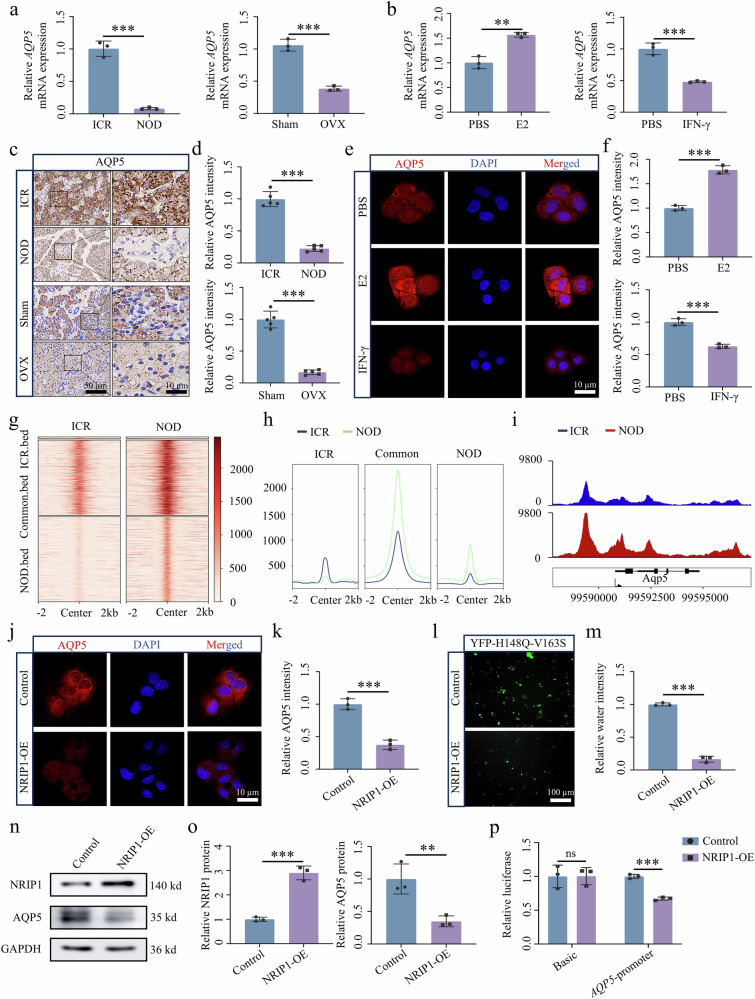
Upregulation of NRIP1 correlates with downregulation of AQP5 in SGECs. **a**, **b** *AQP5* mRNA expression pattern in SMG tissue of NOD and OVX mice (**a**) and estrogen- (E2) or IFN-γ-treated A253 epithelial cells (**b**) (*n* = 3). **c**, **d** AQP5 immunohistochemistry quantification in the SMG of NOD and OVX mice (*n* = 5). **e**, **f** AQP5 immunofluorescence and quantification in E2 or IFN-γ-treated A253 epithelial cells (*n* = 3). **g** The peak center enrichment heat map shows the tag density of ERαBs identified in ICR and NOD SMG tissues. **h** The peak center enrichment plot shows a comparison of average ERα tag counts (in ±2 kb units) at the centers of ERαBs among ICR unique sites, ICR and NOD common sites and NOD unique sites. **i** The occupancy of ERα in AQP5 loci shared between ICR and NOD. **j**–**m** Immunofluorescence staining of AQP5 (**j** and **k**), water secretion (**l** and **m**) and western blot analysis (**n** and **o**) of AQP5 in cells with or without NRIP1 overexpression (*n* = 3). **p** Dual-luciferase reporter assay-based luciferase activity in AQP5 (**n** = 3). Significant difference, ^**^P < 0.01 and ^***^P < 0.001. ERαBs, ERα-binding sites; Sham, sham surgery.

Initially, RT–qPCR was conducted on SMG tissue from the NOD and OVX SjD mouse models. This analysis revealed a notable decrease in *AQP5* expression in the SjD model (Fig. 5a). Furthermore, experiments conducted with SGECs showed increased *AQP5* expression under E2-induced conditions, whereas a decrease was observed under IFN-γ-induced conditions (Fig. 5b). Immunohistochemistry indicated a reduction in AQP5 expression levels in the SjD model group compared with the control group (Fig. 5c, d). In addition, immunofluorescence revealed a significant enhancement in AQP5 staining under E2-induced conditions compared with the PBS group, whereas a notable downregulation was observed under IFN-γ-induced conditions (Fig. 5e, f). Furthermore, immunoprecipitation and deep sequencing analysis of ERα-bound chromatin in SMG tissue from ICR and NOD mice were conducted to complement the expression analysis of target genes. ChIP-seq analysis revealed higher ERα occupancy in the NOD group compared with the ICR group (Fig. 5g, h), with Integrative Genomics Viewer (IGV) results demonstrating increased ERα binding to AQP5 in the NOD group (Fig. 5i).

In addition, immunofluorescence analysis of SGECs overexpressing NRIP1 revealed a significant reduction in intracellular AQP5 expression (Fig. 5j, k). The water permeability of these cells, indicative of cellular water secretion, was assessed using YFP-H148Q-V163S fluorescence, which demonstrated a notable decrease in intracellular water secretion in SGECs overexpressing NRIP1 (Fig. 5l, m). Western blot analysis corroborated these findings, showing consistent changes in AQP5 protein levels (Fig. 5n, o). Moreover, we conducted a dual-luciferase reporter assay, which indicated that the activity of the pGL3-*AQP5*-promoter was significantly diminished in NRIP1-overexpressing SGECs. By contrast, the luciferase activity of pGL3-basic AQP5 remained unchanged following NRIP1 overexpression (Fig. 5p). Collectively, these results provide compelling evidence that NRIP1 downregulates AQP5 gene expression.

### NRIP1–ERα complex binds to ERE sites on the *AQP5* promoter and inhibits AQP5 expression and water secretion

**Fig. 6:**
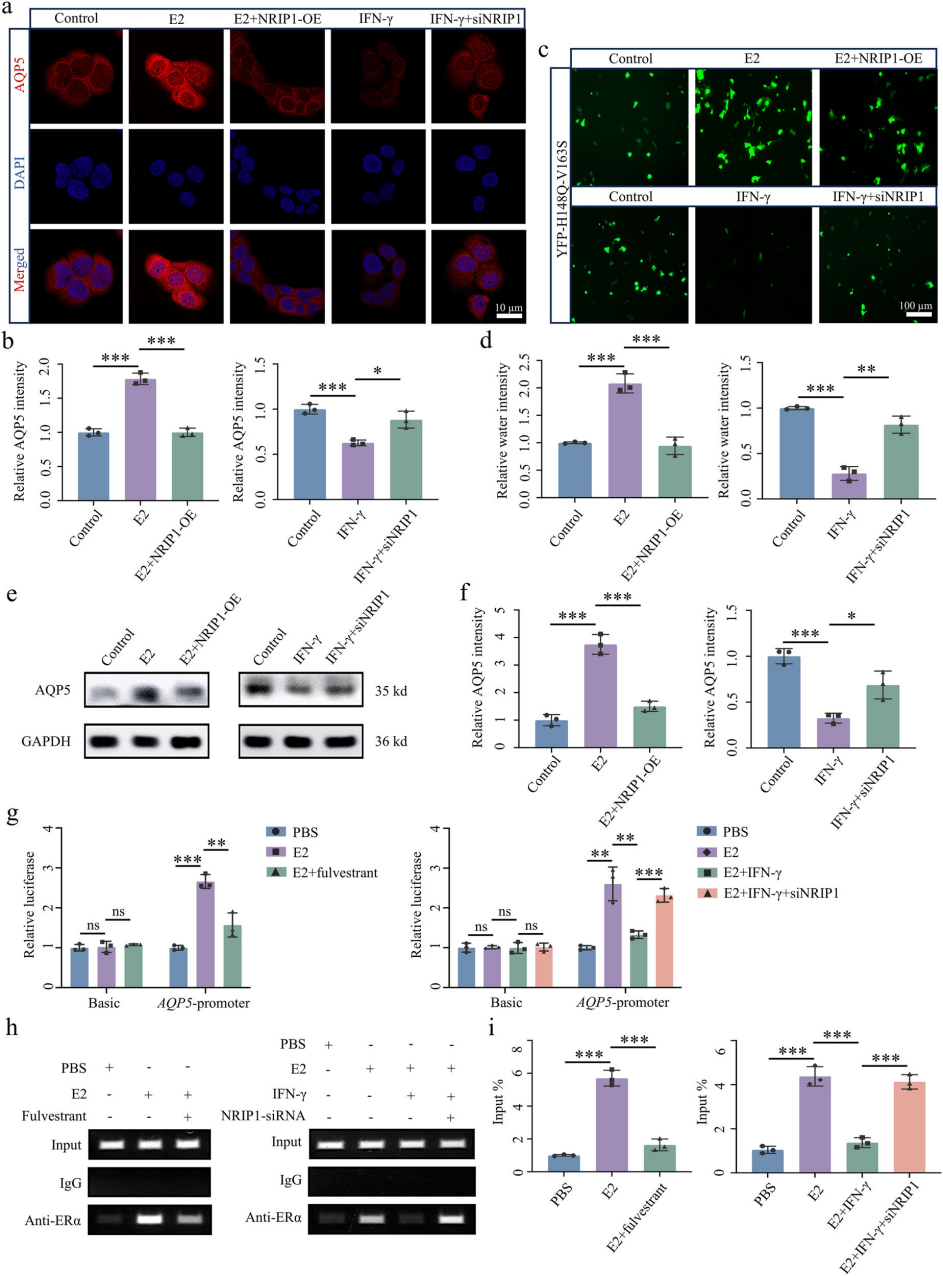
NRIP1–ERα complex binds with ERE sites of *AQP5* promoter to downregulate AQP5 expression. **a**–**f** Immunofluorescence staining (**a** and **b**), water secretion (**c** and **d**) and western blot (**e** and **f**) analysis of AQP5 in NRIP1 overexpressed or NRIP1-knockdown treated A253 epithelial cells in the presence of estrogen (E2) or IFN-γ (*n* = 3). **g** Dual-luciferase reporter assay-based luciferase activity in *AQP5* (*n* = 3). **h**, **i** ChIP assay-based verification of ERE at *AQP5* promoter region in A253 epithelial cells (*n* = 3). Significant difference, ^*^P < 0.05, ^**^P < 0.01 and ^***^P < 0.001. NRIP1-OE, NRIP1 overexpression.

In vitro experiments were conducted on SGECs under conditions simulating SjD to investigate the impact of NRIP1 on AQP5 expression. Immunofluorescence staining revealed that E2 increased AQP5 expression and water secretion. However, E2 was unable to rescue the AQP5 expression and water secretion inhibited by NRIP1 overexpression. By contrast, treatment with IFN-γ suppressed AQP5 expression and water secretion in SGECs, which were partially restored by the knockdown of NRIP1 (Fig. 6a–d). Western blot confirmed changes in AQP5 protein levels (Fig. 6e, f). Furthermore, the dual-luciferase reporter assays demonstrated that E2 enhanced the AQP5 promoter activity, while fulvestrant exhibited a weakening effect. Furthermore, the combination of E2 and IFN-γ resulted in a reduction in promoter activity, which was reversed by the concurrent NRIP1 knockdown (Fig. 6g). These findings indicate that NRIP1 negatively regulates both the gene expression and activity of AQP5.

To investigate the inhibitory effect of the NRIP1–ERα complex on AQP5 gene expression, we conducted ChIP assays to identify the binding site of the NRIP1–ERα complex on the *AQP5* promoter with its sequence obtained from the National Center for Biotechnology Information database. The hTFtarget database was used to predict three high-scoring ERE sites within the promoter: ERE1 (−1,430 to 1,416 base pairs (bp)), ERE2 (−690 to 676 bp) and ERE3 (−478 to 464 bp). Subsequently, primers targeting the highest-scoring ERE sites were designed for ChIP assays that were performed using an ERα antibody (Supplementary Fig. 4a). These results showed that ERα binds to the ERE3 site on the *AQP5* promoter in SGECs, with the binding effect being enhanced by E2 and weakened by fulvestrant. Moreover, the addition of E2 and IFN-γ reduced ERα–ERE binding, which was reversed by simultaneous NRIP1-siRNA transfer (Fig. 6h, i). These findings support our conclusion that the NRIP1–ERα complex binds to the *AQP5* promoter ERE sites, leading to the downregulation of AQP5 expression and saliva secretion in SGECs.

### NRIP1–ERα complex binds to *MYC* promoter to upregulate MYC expression

**Fig. 7:**
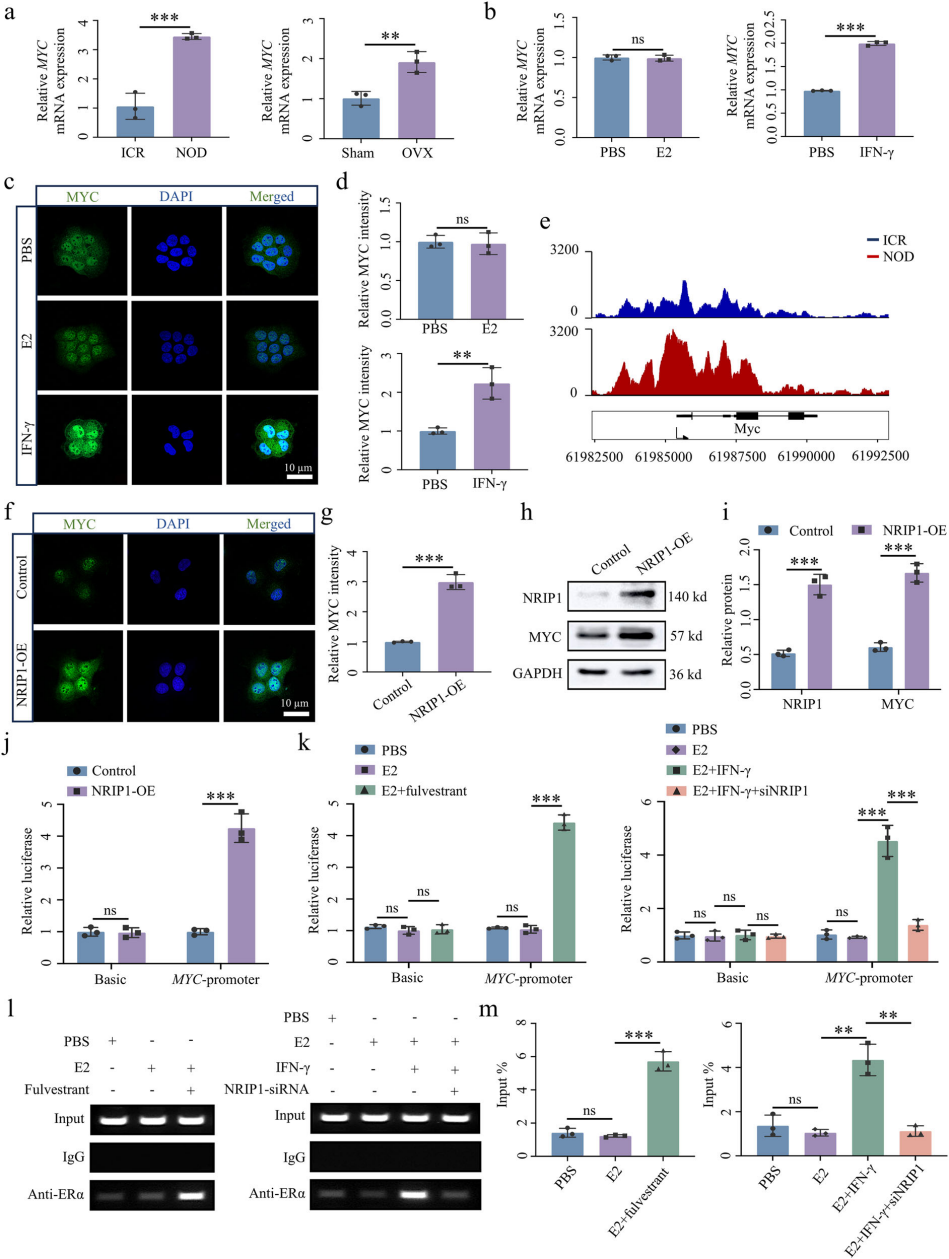
NRIP1–ERα complex binds with ERE sites of *MYC* promoter to upregulate MYC expression. **a**, **b** *MYC* mRNA expression pattern in SMG tissue of NOD and OVX mice (**a**) and estrogen- (E2) or IFN-γ-treated A253 epithelial cells (**b**) (*n* = 3). **c**, **d** MYC immunofluorescence staining in E2- (**c**) or IFN-γ-treated (**d**) A253 epithelial cells (*n* = 3). **e** The occupancy of ERα in MYC loci shared between ICR and NOD. **f**–**i** MYC immunofluorescence staining (**f** and **g**) and western blot assay (**h** and **i**) in NRIP1-overexpressed A253 epithelial cells (*n* = 3). **j**, **k** Dual-luciferase reporter assay detects changes in luciferase activity in *MYC* (*n* = 3). **l**, **m** ChIP assay-based verification of ERE at *MYC* promoter region in A253 epithelial cells (*n* = 3). Significant difference, ^**^P < 0.01 and ^***^P < 0.001. Sham, sham surgery; NRIP1-OE, NRIP1 overexpression.

Initially, we investigated the expression of MYC as a critical downstream target of NRIP1. *MYC* expression was upregulated in the SMG of SjD mouse models and IFN-γ-treated SGECs (Fig. 7a, b). E2 treatment did not alter MYC expression, but IFN-γ treatment robustly upregulated MYC mRNA and protein expression levels (Fig. 7c, d). On the basis of ChIP-seq data analysis, IGV results demonstrated increased ERα binding to the *MYC* promoter in the NOD group (Fig. 7e). Moreover, NRIP1 overexpression robustly upregulated MYC expression (Fig. 7f–i). These results confirmed that the upregulation of MYC in SjD is related to the overexpression of NRIP1.

To investigate the role of the NRIP1–ERα complex in activating *MYC* transcription, we used a dual-luciferase reporter assay. Results showed a significant increase in luciferase activity of the pGL3-*MYC* promoter when transferred with the NRIP1-overexpressing plasmid (Fig. 7j). Interestingly, the addition of E2 did not affect luciferase activity, whereas the simultaneous addition of E2 and fulvestrant led to an increase in the *MYC* promoter activity. Furthermore, the addition of IFN-γ along with E2 increased luciferase activity. Conversely, when E2 and IFN-γ were added with NRIP1-siRNA, luciferase activity was inhibited. Notably, the luciferase activity of pGL3-basic MYC was not impacted by NRIP1 (Fig. 7k). The ChIP assay showed that the binding of the NRIP1–ERα complex to the *MYC* promoter occurred at three ERE sites in the *MYC* promoter sequences, specifically ERE1 (−906 to 896 bp), ERE2 (−692 to 682 bp) and ERE3 (−472 to 462 bp) (Supplementary Fig. 4b).

To verify the effectiveness of the ERE binding site, we designed primers with the highest scores and performed ChIP assays (Supplementary Fig. 4b). The ERα antibody was used to investigate the binding effect of NRIP1–ERα on the *MYC* promoter. As shown in Fig. 7l,m, ERα has a specific binding to the ERE1 site on the *MYC* promoter, and after adding E2, the binding of ERα to ERE did not change significantly. However, after the addition of E2 along with the antagonist fulvestrant, ERα binding to ERE was enhanced. These results indicate that the NRIP1–ERα complex binds to the ERE site of the *MYC* promoter and activates *MYC* transcription. With the addition of IFN-γ at the same time as E2, the binding of ERα to ERE was enhanced, whereas with the addition of E2 and IFN-γ along with NRIP1-siRNA, binding was weakened. These results show that the NRIP1–ERα complex binds to the ERE site of the *MYC* promoter and activates MYC expression.

### MYC impacts SGECs by regulating apoptosis, immunity and metabolism

**Fig. 8:**
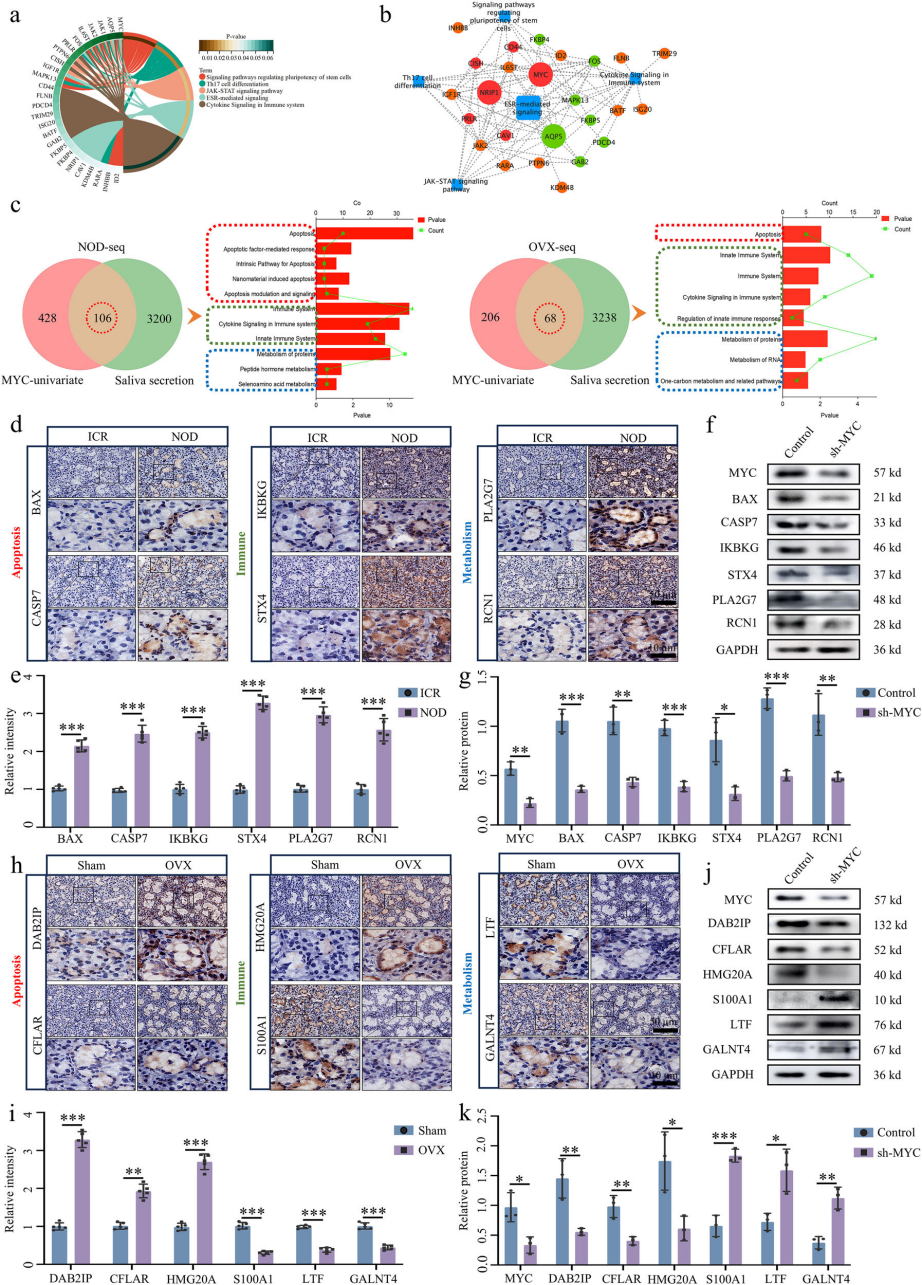
Overexpressed MYC regulates apoptosis, immune regulation and cell metabolism-related transcriptome expression in SGECs during SjD. **a** KEGG enrichment analysis of genes associated with ERα. **b** ERα signal–target gene interaction network. **c** RNA-seq data-based MYC univariate analysis of overlapping DEGs in ICR and NOD mice SMG and KEGG analysis of overlapped DEGs. **d**–**g** Apoptosis, immune regulation and cell metabolism-related protein expression analysis by immunohistochemistry in SMG tissue sections of ICR and NOD mice (**d** and **e**) (*n* = 5) and by western blot analysis in MYC-knockdown A253 epithelial cells (**f** and **g**) (*n* = 3). **h**–**k** Apoptosis, immune regulation and cell metabolism-related protein expression analysis by immunohistochemistry in SMG tissue sections of sham and OVX mice (**h** and **i**) (*n* = 5) and by western blot analysis in MYC-knockdown A253 epithelial cells (**j** and **k**) (*n* = 3). Significant difference, ^*^P < 0.05, ^**^P < 0.01 and ^***^P < 0.001. Sham, sham surgery.

To study events regulated by the downstream target of the NRIP1–ERα signal, RNA-seq from SjD mouse models were further analyzed. First, Kyoto Encyclopedia of Genes and Genomes (KEGG) analysis of annotated ERα signal-related gene alteration showed regulating pluripotency of stem cells, Th17 cell differentiation, JAK–STAT signal, ESR-mediated signal and cytokine signal in the immune system as key signals activated in SjD (Fig. 8a). Next, we established a regulatory network between these signal-annotated genes and signals (Fig. 8b). MYC and AQP5 are at the core of the network as coregulatory genes for signal, with the correlation between NRIP1 and MYC and AQP5. These results suggest that MYC and AQP5 are key altered genes regulated by the ERα signal, and this alteration is tightly correlated with changes in NRIP1.

On the basis of RNA-seq from two SjD mouse models, we performed a univariate analysis of MYC (selected DEGs associated with saliva secretion) and performed a KEGG-annotated analysis of these DEGs. Downstream events regulated by MYC mostly involved apoptosis, immunity and metabolic pathways (Fig. 8c). We then selected the top two in apoptosis (NOD-seq, BAX and CASP7; OVX-seq, DAB2IP and CFLAR) and metabolic (NOD-seq: PLA2G7 and RCN1; OVX-seq: LTF and GALNT4) pathway annotated genes as core targets. As SjD is an autoimmune disease, we selected co-expressed genes related to immune pathway annotation and antigen presentation signals as core targets (NOD-seq: IKBKG and STX4; OVX-seq: HMG20A and S100A1) (Supplementary Fig. 4c). Protein expression levels of these core targets were significantly changed in SjD groups (Fig. 8d, e, h, i).

By transferring sh-MYC plasmids into A253 epithelial cells, we detected changes in MYC target protein expression by western blot. This showed significant differences in expression levels in the sh-MYC group compared with the vector group. Specifically, the expression of BAX, CASP7, DAB2IP, CFLAR, IKBKG, STX4, HMG20A, PLA2G7 and RCN1 was decreased, whereas the expression of S100A1, LTF and GALNT4 was increased (Fig. 8f, g, j, k). These findings were consistent with the immunohistochemistry results obtained from tissue sections. The above results indicate that the upregulated MYC in SjD promotes apoptosis and disrupts immune regulation and cell metabolism, which may aggravate SjD pathogenicity. These results provide a new direction for further in-depth studies into the mechanism of SGEC dysfunction in SjD.

### Computational simulations suggest that NRIP1 competitively inhibits the E2–ERα signal

**Fig. 9:**
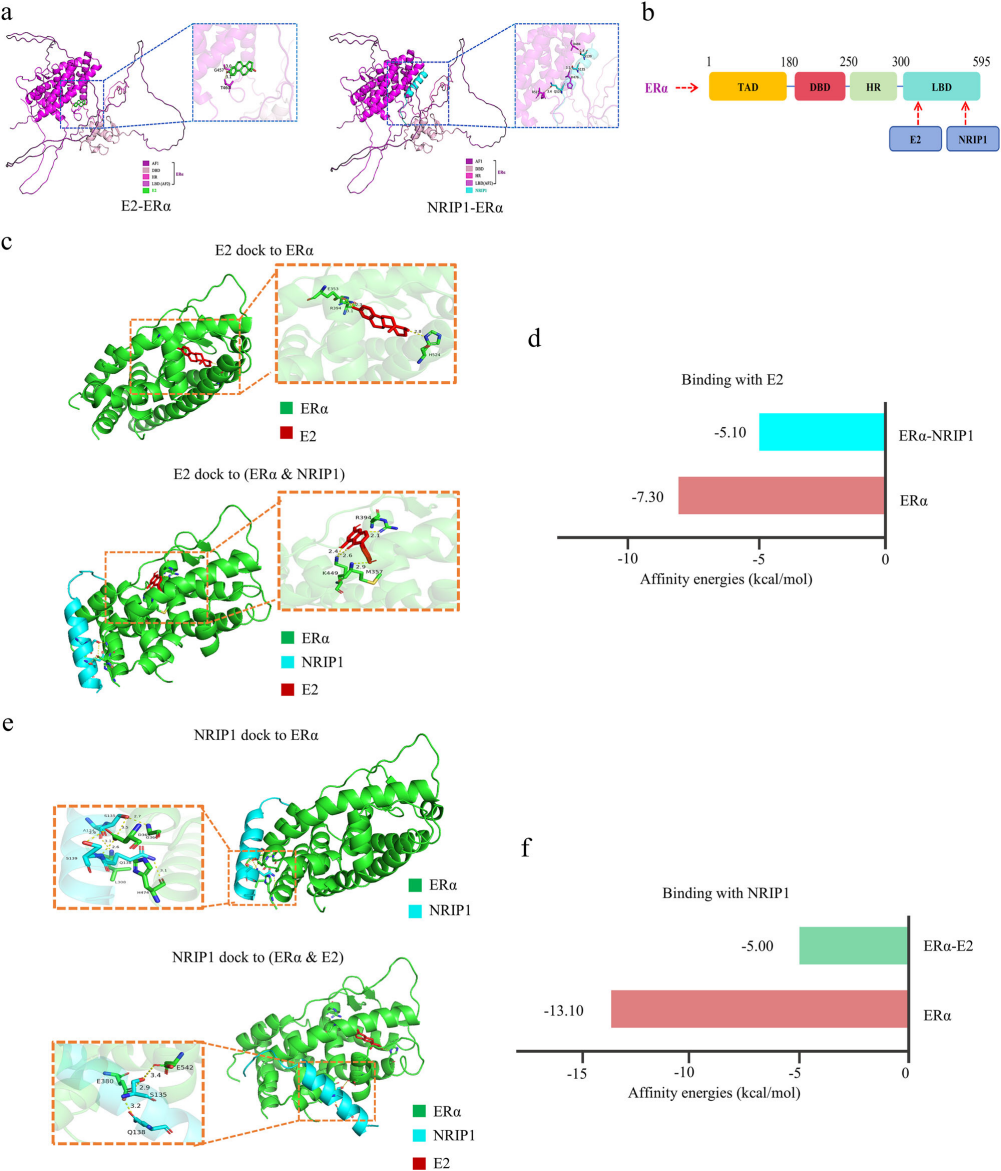
NRIP1 may act by competitively inhibiting E2–ERα signal. **a** Schematic diagram of molecular docking of E2 and NRIP1 with ERα, respectively. **b** Both E2 and NRIP1 bind to the LBD region of ERα. **c** Schematic diagram of molecular docking of E2 with ERα; E2 enters the inner amino acid pocket of ERα protein; E2 binds to the ERα and NRIP1 complex and cannot enter the interior of the ERα protein but binds to the protein surface. **d** The binding free energy of E2 and ERα is −7.30 kcal/mol, while the binding free energy of E2 and NRIP1–ERα complex is −5.10 kcal/mol. **e** Molecular docking diagram of NRIP1 and ERα. NRIP1 binds to one side of ERα, and several amino acids participate in the binding of the two. Molecular docking diagram of NRIP1 and E2–ERα complex. NRIP1 binds to the surface of the E2–ERα complex, and fewer amino acids are involved in the binding of the two. **f** The binding free energy of NRIP1 with ERα is −13.10 kcal/mol, while the binding free energy of NRIP1 with the E2–ERα complex is −5.00 kcal/mol.

To evaluate the differential roles of NRIP1–ERα and E2–ERα signal in promoter transcriptional regulation, molecular docking analysis using AlphaFold3 was conducted. The analysis revealed that both E2 and NRIP1 have specific binding regions within the molecular structure of the ERα protein (Fig. 9a). Notably, both E2 and NRIP1 interact with the ligand-binding domain (LBD) of ERα, indicating a possible competitive binding between the two (Fig. 9b). This finding offers an alternative perspective on the interplay between NRIP1 and the E2–ERα signal.

To elucidate the potential mechanism by which NRIP1 is involved in the ERα signal, we conducted molecular docking and energy analysis using Autodock, focusing on the cobinding region of E2–ERα and NRIP1–ERα (LBD of ERα). Our key findings are as follows. First, E2 was used to model and analyze molecular docking. The results indicated that the binding free energy of E2 to ERα was −7.30 kcal/mol, whereas the binding free energy of the E2–NRIP1–ERα complex was −5.10 kcal/mol. This implies that E2 can effectively inhibit the interaction between NRIP1 and ERα (Fig. 9d). Second, we used Autodock molecular docking to simulate the alterations in the binding region caused by the competitive interaction of E2 and NRIP1 with ERα. Notably, E2 is situated within the amino acid pocket of ERα (Fig. 9c). However, when ERα associates with NRIP1 to form the ERα–NRIP1 complex, E2 is unable to occupy the amino acid pocket and is displaced to the exterior of the ERα protein (Fig. 9c). Consequently, the binding energy is significantly diminished (Fig. 9d), indicating that the binding of NRIP1 to ERα inhibits the binding of E2 to ERα. Third, NRIP1 was used for molecular docking modeling and analysis. The findings revealed that the binding free energy of NRIP1 to ERα alone was −13.10 kcal/mol, whereas the binding free energy of NRIP1 to the E2–ERα complex was considerably lower at −5.00 kcal/mol (Fig. 9f). This discrepancy suggests that the E2–ERα complex has significantly diminished its binding capacity for NRIP1, indicating a competitive interaction between E2 and NRIP1 for ERα binding. As shown in the molecular docking diagram, NRIP1 binds to one side of ERα, engaging multiple amino acids in this interaction (Fig. 9e). By contrast, when NRIP1 interacts with the E2–ERα complex, it binds to the surface of the complex, involving fewer amino acids in the binding process (Fig. 9e). Furthermore, the interface observed when ERα binds to E2 and subsequently to NRIP1 differs from that when ERα binds directly to NRIP1 (Fig. 9e, left), with a substantial energy difference noted (Fig. 9f). This indicates that the E2–ERα complex has largely lost its capacity to bind NRIP1. Collectively, these results predict the mutual exclusivity between the NRIP1–ERα complex and the E2–ERα complex, providing structural evidence that NRIP1 may competitively inhibit the E2–ERα signal.

## Discussion

E2 is a key regulator of SG function and its deficiency in SjD inactivates downstream ERα signal that affects SG function^18,19,43^. However, regulation of the ERα signal in the context of inflammation and reduced E2 levels in SjD has been unclear. This present study revealed a robust expression of NRIP1 in SGECs in SjD. NRIP1 has a dual function of transcriptional inhibition and activation, which has been reported to regulate ERα signal in the mammary gland and female reproductive system^27,44^. Here, we demonstrate that upregulated NRIP1 in SjD directly interacts with ERα and inhibits E2–ERα interactions in SGECs. Furthermore, the NRIP1–ERα complex downregulates AQP5 expression and upregulates MYC expression in SGECs by binding to ERE sites in the promoters of *AQP5* and *MYC*. The downregulation of AQP5 is associated with reduced saliva secretion, while the upregulation of MYC is linked to SGEC apoptosis and disrupted immune response and cell metabolism. Experiments conducted with NRIP1 KO mice revealed a reversal of SjD symptoms. In addition, computer simulations suggest that NRIP1 may exert a competitive inhibitory effect on the E2–ERα signal. Our research highlights the distinction between physiological E2–ERα signal and pathological NRIP1–ERα signal in regulating the survival and function of SGECs. Overall, our findings suggest that elevated NRIP1–ERα signal in SjD serves as central pathological signal that downregulates AQP5 expression and upregulates MYC expression in SGECs, ultimately leading to SG dysfunction.

The E2–ERα signal has been reported to inhibit apoptosis of SGECs, maintain glandular structure and avoid auto-inflammation^45,46^. In SjD, E2 levels are reduced and the E2–ERα signal is inactivated in SGECs^18,47^. Here, we used RNA-seq of the SMG from SjD mouse models, and, through GSEA, we identified the ERα signal as the most critical disease pathway in both SjD models. This finding further confirms the close correlation between ERα signal imbalance and the progression of SjD. We identified important core regulators of the ERα signal through co-expression screening, which were subsequently validated using in vitro and in vivo studies, clinical data and clinical samples. Specifically, NRIP1 was found to be highly expressed in the inflammatory pathological environment and under conditions of estrogen decline of SjD. However, the upstream regulatory mechanisms governing NRIP1 expression remain unclear. Our previous research found that IFN-γ, a key proinflammatory factor in SjD, regulates gene transcription by activating the JAK–STAT pathway^36^, which aligns with existing literature^48^. Intriguingly, studies in macrophages indicate that miR-499 activates the JAK–STAT pathway by targeting and inhibiting NRIP1^49^. This suggests a paradigm where IFN-γ could potentially mediate NRIP1 transcription to engage STAT-dependent pathways. Alternatively, the postmenopausal decline in estrogen may derepress NRIP1 transcription. Estrogen receptors typically recruit corepressor complexes (for example, NCoR–SMRT) to silence target genes^50,51^. Estrogen deficiency could attenuate this repression or indirectly elevate NRIP1 via other transcription factors such as NF-κB^52,53^. Alterations in the post-transcriptional regulation of NRIP1 in SjD also warrant investigation. These perspectives offer plausible avenues for elucidating NRIP1 upregulation in the disease. Our molecular docking and correlation analyses demonstrated a robust interaction between NRIP1 and ERα. Furthermore, scRNA-seq analysis of SjD revealed that NRIP1 and ERα were highly co-expressed in SjD cells during the disease state. Notably, significant co-expression of NRIP1 and ERα was observed in SjD mouse models, clinical samples and cell models. Interestingly, the downstream target genes regulated by NRIP1 and ERα were found to be highly consistent in SjD mouse models, suggesting a concert function in the context of SjD.

In addition, we generated *Nrip1*-KO/OVX mice to evaluate the role of NRIP1 in the development of SjD. Our findings indicate that the knockdown of NRIP1 effectively reverses the SjD phenotype. Furthermore, reduced levels of NRIP1 corresponded with decreased anti-SSA and anti-SSB antibodies in the serum of OVX mice, suggesting that *NRIP1* gene KO may alleviate postovariectomy autoimmune symptoms. Notably, the evaluation of changes in the expression levels of NRIP1 and ERα in SMG tissue revealed that *NRIP1* gene KO effectively restored the loss of ERα signal expression following ovariectomy. These results suggest that OVX mice exhibit a SjD phenotype and that the SjD phenotype in mice following *NRIP1* gene KO is significantly improved, indicating that high expression of NRIP1 plays a crucial role in the onset and progression of SjD.

Nautiyal et al. reported that the NRIP1–ERα complex regulates amphiregulin (Areg), the progesterone receptor (Pgr) and signal transducer and activator of transcription 5a (Stat5a) expression that influence key mitogenic pathways related to normal mammary gland development^40^. Our results showed that AQP5 and MYC are downstream targets of the NRIP1–ERα complex in SGECs. The interaction with E2–ERα activates SGEC survival and saliva secretion by inducing AQP5 expression^54^. AQP5 is the key protein that regulates SGEC function, including cell survival and saliva secretion, and reduced expression of AQP5 in SGECs is common in SjD^11^. Here, we discovered that there is reduced expression of AQP5 in SG of SjD mice and IFN-γ-treated SGECs, whereas E2 treatment promotes AQP5 expression in SGECs. Moreover, NRIP1 overexpression downregulates AQP5 expression and saliva secretion in SGECs, whereas E2 treatment cannot rescue NRIP1 overexpression-inhibited AQP5 expression. This suggests that NRIP1 is a competitive inhibitor of E2 attenuating E2– ERα interactions. Augereau et al. reported the negative regulatory role of NRIP1 in E2–ERα signal^35^. The NRIP1–estrogen receptor interaction inhibits AP-1-dependent downstream transcription. Our ChIP-seq analysis showed that the NOD group had a higher occupancy of ERα and increased binding of ERα to AQP5. In addition, we found that the NRIP1–ERα complex inhibits AQP5 expression by directly binding to ERE sites on the AQP5 promoter. These results indicate that NRIP1 exerts a negative regulatory role in ERα-mediated AQP5 expression that contributes to SjD.

NRIP1 can not only co-activate but it can also co-inhibit the transcriptional process. NRIP1 has been reported to co-activate gene transcription related to various biological activities, including inflammation, mammary gland development and ovulation^27,44^. As our results indicate, *MYC* is another target gene of the NRIP1–ERα complex in SGECs in SjD. Our RNA-seq, ChIP-seq, in vitro and in vivo studies and SjD clinical samples all demonstrate that in contrast to the AQP5, there is robust upregulation of MYC in SGECs in SjD. The proto-oncogene *MYC* is essential for cell reprogramming, chromatin epiregulation, cell fate determination and cell structure remodeling^55^. MYC also regulates cell apoptosis, tumorogenesis and aging^56,57^. We found that E2 treatment did not affect MYC expression, whereas IFN-γ treatment robustly upregulates MYC expression in SGECs. Moreover, NRIP1 overexpression upregulated MYC expression mainly by targeting ERE sites on the MYC promoter. Reduced levels of MYC have been reported to increase longevity and enhance health span^58^. As SjD is an aging-related disease, upregulated MYC in SjD SGs might influence SG function. Here, we found that upregulated MYC in SjD SGECs promotes the expression of pro-apoptotic factors, that is, BAX, CASP7, DAB2IP and CFLAR. BAX^59^ and CFLAR^60^ have been reported to promote SGEC apoptosis, and CASP7 and DAB2IP have been reported to promote apoptosis in cancer^61,62^. Upregulated MYC also altered the expression of cell metabolism regulation markers, that is, upregulation of PLA2G7 and RCN1 and downregulation of LTF and GALNT4. Spadaro et al. reported the regulatory role of PLA2G7 in adipose tissue immune metabolism^61^. RCN1 regulates tumor progression^63^, and LTF and GALNT4 regulate cell growth^64,65^. As SjD is an autoimmune disease, disruption of immune regulation plays a crucial role in disease initiation and progression^66^. Moreover, upregulated MYC in SjD SGECs altered immune regulation marker expression, that is, upregulation of IKBKG, STX4 and HMG20A and downregulation of S100A1. Upregulated IKBKG^67^ and STX4^68^ have been shown to play a crucial role in the immunomodulation of SjD, thereby accelerating disease progression. HMG20A has been reported to regulate the inflammatory response in myocardial fibrosis^69^, and S100A1 has been found to participate in hypoxia-induced cardiomyocyte inflammation^70^. These results suggest that activated NRIP1–ERα signal in SjD SGECs upregulates MYC expression, which further regulates apoptosis, cell metabolism and immune regulation to exacerbate disease pathogenicity.

Finally, we evaluated the differential roles of the NRIP1–ERα signal and E2–ERα signal in the regulation of promoter transcription. Notably, both E2 and NRIP1 interact with the LBD of ERα, indicating a potential competitive binding relationship between the two. This discovery offers a new perspective on the possible interplay between NRIP1 and E2–ERα signal. Furthermore, computer simulations predict a mutual repulsion between the NRIP1–ERα signal and the E2–ERα signal, providing structural evidence that NRIP1 may competitively inhibit E2–ERα signal. Overall, our study suggests that targeting the NRIP1–ERα signal axis could represent a therapeutic approach for treating SjD. However, the limited research on the mechanisms underlying NRIP1 upregulation during SjD remains a significant gap in current literature.

## Supplementary information

Supplementary Information
