# Supplementary material for: NRIP1 disrupts ERα signal in Sjögren’s disease via AQP5 suppression and MYC-driven salivary dysfunction
Source: Exp Mol Med. 2026 Mar 13;58(3):898–916. doi: 10.1038/s12276-026-01671-w (PMC13049167; doi:10.1038/s12276-026-01671-w)
Supplement: Supplementary file 1 — Supplementary Information [file 12276_2026_1671_MOESM1_ESM.pdf]

Supplementary file

NRIP1 disrupts ERα signal in Sjögren's disease via AQP5 suppression and MYC-driven salivary dysfunction

Bo Chen[1#], Janak L. Pathak[1#], Xiuni Qin[2], Xueyang Li[3], Tianjiao Mao[1], Xi Chen[1], Wei Wei[4], Nobumoto Watanabe[5], Lijing Wang[1], Kevin H Mayo[6], Jun Di[7*], Yongliang Huo[8*], Xiaomeng Li[9], Jiang Li[1*]

1 School and Hospital of Stomatology, Guangdong Engineering Research Center of Oral Restoration and Reconstruction, Guangzhou Key Laboratory of Basic and Applied Research of Oral Regenerative Medicine, Guangzhou Medical University, Guangzhou, 510182, China.
2 Guangzhou Concord Cancer Center, Guangzhou, Guangdong, China.
3 Department of Dentistry and Endodontics, Affiliated Stomatology Hospital of Guangzhou Medical University, Guangzhou, Guangdong, China.
4 Hospital of Stomatology, Jilin University, Changchun, Jilin, China.
5 Chemical Resource Development Research Unit, RIKEN Center for Sustainable Resource Science, Saitama 351-0198, Japan.
6 Department of Biochemistry, Molecular Biology & Biophysics, University of Minnesota Health Sciences Center, 6-155 Jackson Hall, Minneapolis, MN 55455, USA.
7 Beijing University of Chinese Medicine Hospital in Shenzhen (Longgang), #1 Dayun Road Longgang District, Shenzhen, Guangdong Province, China.
8 Guangzhou Municipal and Guangdong Provincial Key Laboratory of Protein Modification and Degradation, School of Basic Medical Sciences, Guangzhou Medical University, Guangzhou, Guangdong, China.
9 KingMed School of Laboratory Medicine, Guangzhou Medical University, Guangzhou, Guangdong, China.

# Bo Chen and Janak L. Pathak have equal contributions to this paper and share the first authorship.

***Corresponding authors**
***Jiang Li (Professor)**
Guangdong Engineering Research Center of Oral Restoration and Reconstruction, Affiliated Stomatology Hospital of Guangzhou Medical University, Guangzhou, China.
#195 Dongfeng West Road, Guangzhou, Guangdong, China, 510182
Phone: (020) 8050 0893
E-mail: ljiang@gzhmu.edu.cn

***Jun Di (Professor)**
Email: dijun1991@163.com

***Yongliang Huo (Professor)**
Email: yongliang.huo@gzhmu.edu.cn

Supplementary Table 1. The information of microarray datasets.

GEO series	Platform	Samples no.	
		Ctrl	SjD
GSE143153	GPL13607	15	17
GSE97614	GPL6244	3	9
GSE127952	GPL20995	6	8

Supplementary Table 2. The 2016 ACR/EULAR criteria.

Series	Item	Weight
1	Labial salivary gland with focal lymphocytic sialadenitis and focus score of > 1 foci/4 mm^2	3
2	Anti-Ro positive	1
3	Ocular staining score > 5 in at least one eye	1
4	Schirmer's test < 5 mm/5 min in at least one eye	1
5	Unstimulated whole saliva flow rate < 0.1 ml/min	1

An individual with a score of > 4 is classified as having primary SjD.

Supplementary Table 3. The detailed clinical data of the patients with SjD included in this study.

Clinical characteristic, n/N (%)	SjD (n = 5)
Age (years)#	45.8 (6.69)
Female	5/5 (100.00)
Schirmer's test	4/5 (80.00)
UWS flow	4/5 (80.00)
Focus score of ≥1	5/5 (100.00)
Anti-SSA/Ro positive	4/5 (80.00)
Anti-SSB/La positive	2/5 (40.00)
ANA positive	5/5 (100.00)
RF (IU/mL)*	161 (95, 219)
Neutrophils (*10^9/L)*	3.52 (3.47, 3.61)
Lymphocytes (*10^9/L)*	1.38 (1.26, 2.03)
Thrombocytes (*10^9/L)*	333 (231, 338)
Hemoglobin (g/L)*	110 (109, 116)
IgG (g/L)#	25.98 (7.91)
IgM (g/L)*	1.92 (1.02, 1.96)
IgA (g/L)#	3.80 (1.88)
C3 (mg/L)#	1.13 (0.16)
C4 (mg/L)#	0.28 (0.11)
CRP (mg/L)*	8.7 (7.2, 10.8)

#: mean (standard deviation); *: median (interquartile range); RF: rheumatoid factor; Focus score ≥1: Labial salivary gland with focal lymphocytic sialadenitis and focus score of ≥1 foci per 4 mm^2; Schirmer's test positive: ≤5 mm/5 min; UWS flow positive: whole unstimulated saliva flow≤0.1 mL/min; CRP: C-reactive protein.

Supplementary Table 4. The sequences of plasmids used in this study.

Group	Gene	Target sequences (5′–3′)
si-NRIP1 sequences	si-NC	GCATTTCCCACCTGTCAAAGT
	si-NRIP1 #1	GCTAAACTCACACCAGAAAGT
	si-NRIP1 #2	GCATTTAGTAGTCAACCAACA
	si-NRIP1 #3	GCAGAAACGAAAGCTTCTTTC
	si-NRIP1 #4	GCGCAAATGGAGAAGTTTATG
sh-MYC sequences	sh-NC	GGAAACGACGAGAACAGTTGA
	sh-MYC #1	GCTTCACCAACAGGAACTATG
	sh-MYC #2	GCTTGTACCTGCAGGATCTGA
	sh-MYC #3	GAGAATGTCAAGAGGCGAACA
	sh-MYC #4	GGAAGAAATCGATGTTGTTTC

Supplementary Table 5. List of primers used in this study.

Primer Name	Forward primer (5′–3′)	Reverse primer (5′–3′)
ERα	CCTCCTCATCCTCTCCCACATCAG	GCATCTCCAGCAGCAGGTCATAG
NRIP1	TGAGTGAACCGTTGTCATGTGCTG	TTAGGTGAGGTGGCAGGACTAGC
AQP5	GGCTGCCATCCTTTACTTCTACCTG	GCTCCTCCCAGTCCTCGTCAG
MYC	AGCAGCGACTCTGAGGAGGAAC	TCCAGCAGAAGGTGATCCAGACTC
β-actin	ATCACTATTGGCAACGAGCGGTTC	CAGCACTGTGTTGGCATAGAGGTC

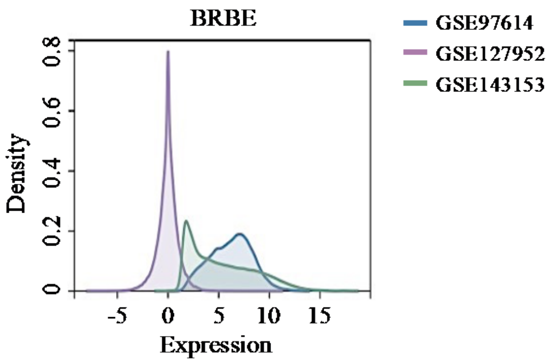

Supplementary Fig 1. Density maps illustrate the impact of integrating multiple SjD salivary gland transcriptome datasets prior to the removal of batch effects. BRBE, before remove batch effect.

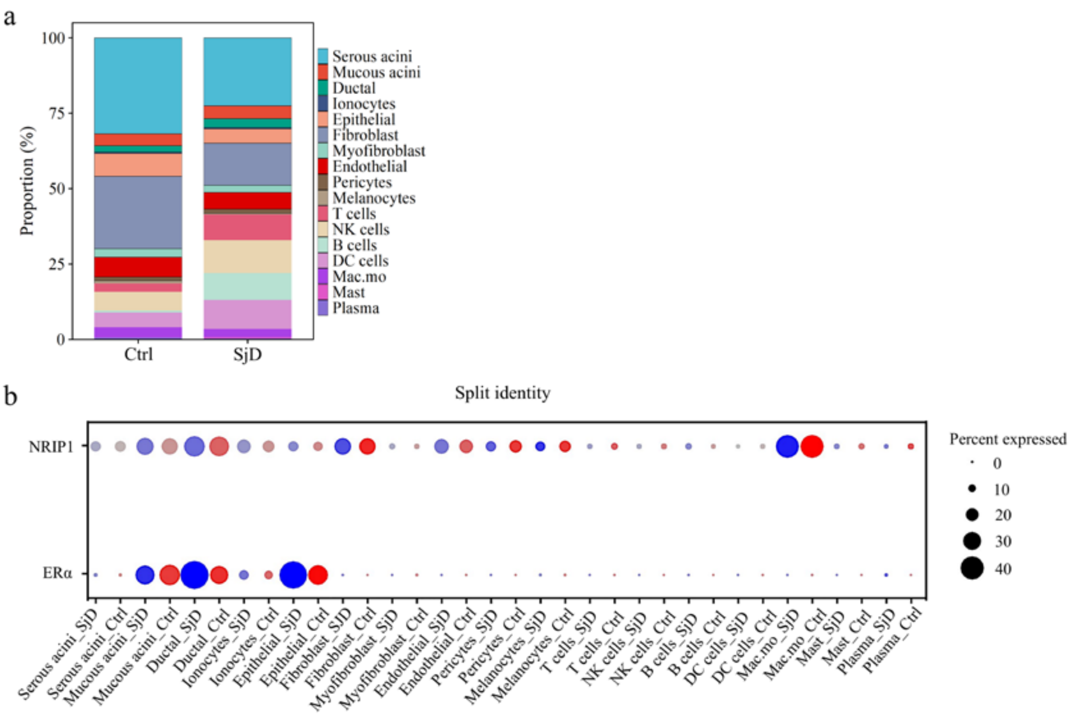

Supplementary Fig 2. Analysis of scRNA-seq data from SjD. (a) Cellular composition and the number of cells of all cell types across the various sample types examined in this study. (b) Dot plot illustrating the expression levels of the NRIP1 and ERα genes in each cell subset.

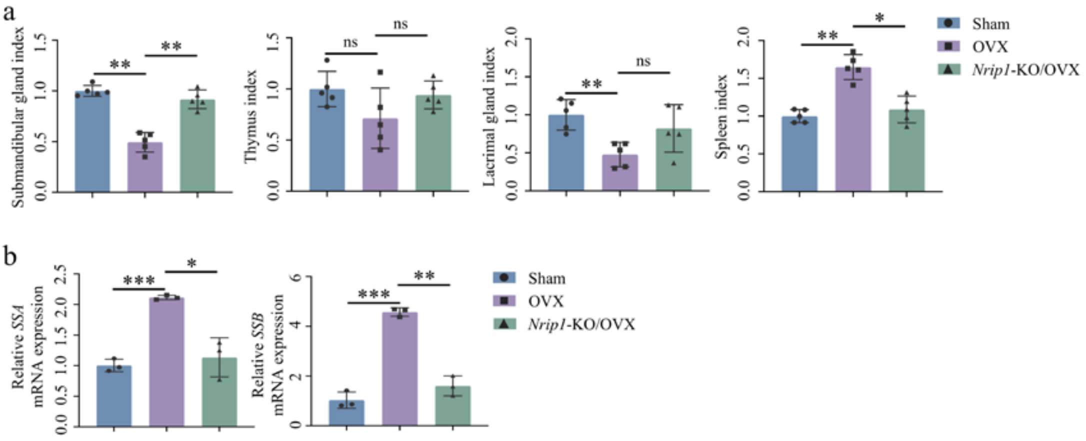

Supplementary Fig 3. Determination of indicators in mouse models. (a) The organ index statistics of each group of mice (n = 5). (b) *SSA* and *SSB* mRNA expression pattern in SG tissue of mice (n = 3). Significant difference, **p* < 0.05, ***p* < 0.01, and ****p* < 0.001. Sham, sham surgery; OVX, ovariectomy.

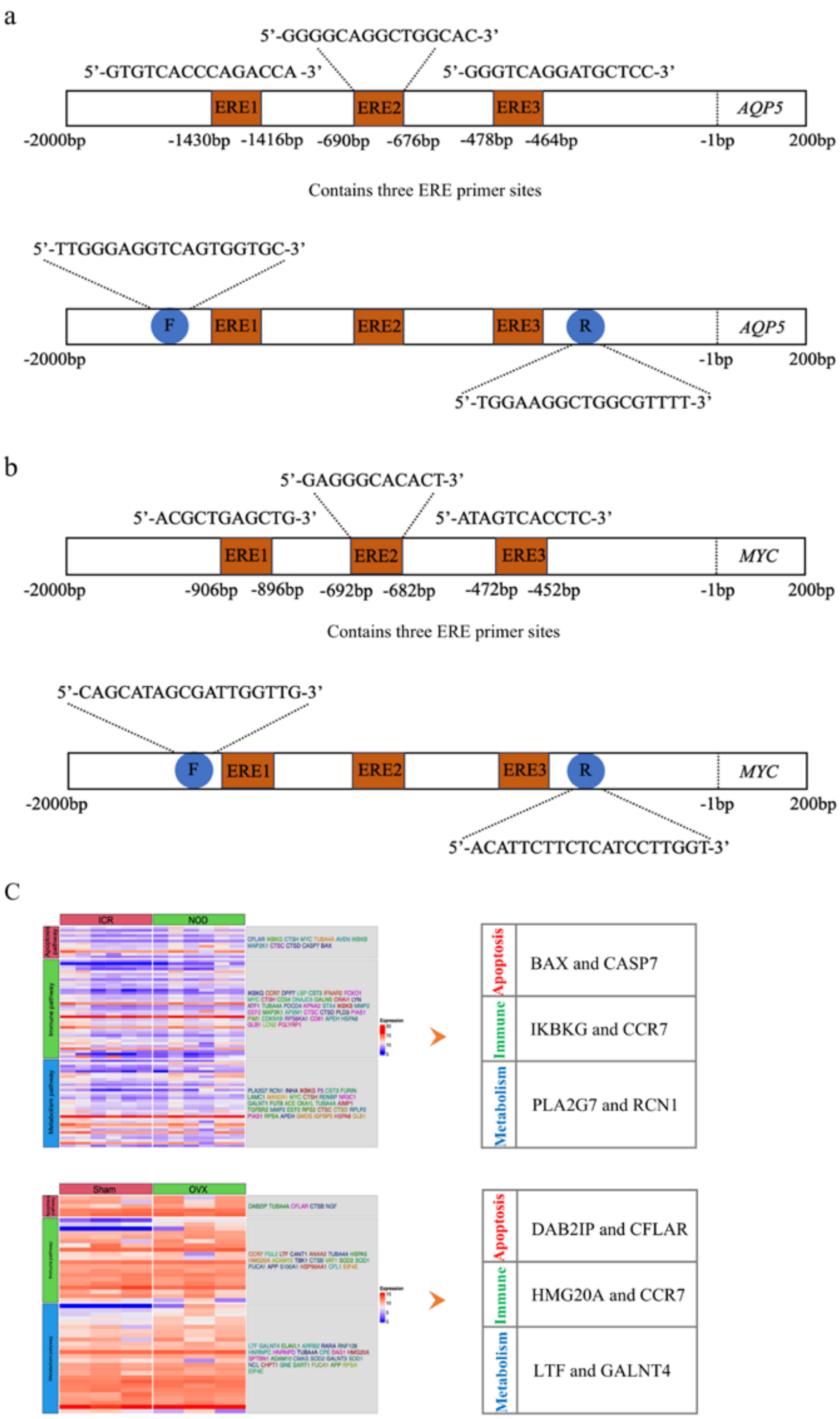

Supplementary Fig 4. Prediction of ERE sites in the *AQP5* and *MYC* promoter regions, and identification of core genes. (a) The predicted *AQP5* promoter region contains the ERE sequence determined by the hTFtarget database, and primer design for ChIP assay targeting the ERE in the *AQP5* promoter sequences. (b) The predicted *MYC* promoter region contains the ERE sequence determined by the hTFtarget database, and primer design for ChIP assay targeting the ERE in the *MYC* promoter sequences. (c) Apoptosis, immune regulation, and cell

metabolism-related altered genes in ICR vs NOD and Sham vs OVX mice. ICR, Institute of Cancer Research; NOD, non-obese diabetic; Sham, sham surgery; OVX, ovariectomy; ERE, estrogen response element.
